# Methylation status of genes escaping from X-chromosome inactivation in patients with X-chromosome rearrangements

**DOI:** 10.1186/s13148-021-01121-6

**Published:** 2021-06-30

**Authors:** Sayaka Kawashima, Atsushi Hattori, Erina Suzuki, Keiko Matsubara, Machiko Toki, Rika Kosaki, Yukihiro Hasegawa, Kazuhiko Nakabayashi, Maki Fukami, Masayo Kagami

**Affiliations:** 1 Department of Molecular Endocrinology, National Research Institute for Child Health and Development, 2-10-1 Okura, Setagaya-ku, Tokyo 157-8535 Japan; 2 Department of Pediatrics, Tohoku University School of Medicine, 1-1 Seiryomachi, Aobaku, Sendai, Miyagi 980-8574 Japan; 3 Department of Pediatrics, Hiratsuka City Hospital, 1-19-1 Minamihara, Hiratsuka, Kanagawa 254-0065 Japan; 4 Department of Pediatrics, Keio University School of Medicine, 35 Shinanomachi, Shinjuku-ku, Tokyo 160-8582 Japan; 5 Division of Medical Genetics, National Center for Child Health and Development, 2-10-1 Okura, Setagaya-ku, Tokyo 157-8535 Japan; 6 Division of Endocrinology and Metabolism, Tokyo Metropolitan Children’s Medical Center, 2-8-29 Musashidai, Fuchu, Tokyo 183-8561 Japan; 7 Department of Maternal Fetal Biology, National Research Institute for Child Health and Development, 2-10-1 Okura, Setagaya-ku, Tokyo 157-8535 Japan

**Keywords:** X-chromosome inactivation, Escape gene, X-chromosome rearrangement

## Abstract

**Background:**

X-chromosome inactivation (XCI) is a mechanism in which one of two X chromosomes in females is randomly inactivated in order to compensate for imbalance of gene dosage between sexes. However, about 15% of genes on the inactivated X chromosome (Xi) escape from XCI. The methylation level of the promoter region of the escape gene is lower than that of the inactivated genes. *Dxz4* and/or *Firre* have critical roles for forming the three-dimensional (3D) structure of Xi. In mice, disrupting the 3D structure of Xi by deleting both *Dxz4* and *Firre* genes led to changing of the escape genes list. To estimate the impact for escape genes by X-chromosome rearrangements, including *DXZ4* and *FIRRE,* we examined the methylation status of escape gene promoters in patients with various X-chromosome rearrangements.

**Results:**

To detect the breakpoints, we first performed array-based comparative genomic hybridization and whole-genome sequencing in four patients with X-chromosome rearrangements. Subsequently, we conducted array-based methylation analysis and reduced representation bisulfite sequencing in the four patients with X-chromosome rearrangements and controls. Of genes reported as escape genes by gene expression analysis using human hybrid cells in a previous study, 32 genes showed hypomethylation of the promoter region in both male controls and female controls. Three patients with X-chromosome rearrangements had no escape genes with abnormal methylation of the promoter region. One of four patients with the most complicated rearrangements exhibited abnormal methylation in three escape genes. Furthermore, in the patient with the deletion of the *FIRRE* gene and the duplication of *DXZ4,* most escape genes remained hypomethylated.

**Conclusion:**

X-chromosome rearrangements are unlikely to affect the methylation status of the promoter regions of escape genes, except for a specific case with highly complex rearrangements, including the deletion of the *FIRRE* gene and the duplication of *DXZ4*.

**Supplementary Information:**

The online version contains supplementary material available at 10.1186/s13148-021-01121-6.

## Background

X-chromosome inactivation (XCI) is a mechanism in which one of two X chromosomes in females is randomly inactivated to compensate for the imbalance of gene dosage between males and females [1]. However, approximately 15% of genes on the inactivated X chromosome (Xi) escape from XCI (called “escape genes”) that show biallelic expression in humans [2]. Most escape genes are on Xp [2], including pseudoautosomal region 1 (PAR1), which has a homologous sequence at the distal end of Yp. All genes in PAR1 escape from XCI and are expressed from both Xi and activated X chromosome (Xa) [2]. In addition, about 20% to 30% of genes on Xi exhibit different escape status from XCI among individuals or tissues (variable escape gene) [2–4]. XCI begins from Xi-specific expression of long non-coding RNA, namely *XIST* [5]. *XIST* RNA spreads in *cis* along the X chromosome and causes inactivation [5]. XCI results in hypermethylation of the promoter regions of inactivated genes [4]. On the other hand, escape genes on Xi have hypomethylated promoter regions [4]. The differences between the processes of XCI in humans and mice [6] lead to difficulty in clarifying the mechanisms of XCI and escape in humans. Furthermore, XCI mosaicism caused by random inactivation has made it complicated to study escape genes [7]. To overcome these issues, previous studies examined allele-specific expression using somatic cell hybrids [2], compared methylation levels of CpG on the X chromosome using methylation arrays in both sexes [4], and investigated expression of escape genes in somatic cells using single-cell RNA sequencing [8].

The mechanism of escape remains to be revealed. Previous study suggested that surrounding sequences of escape genes contained an intrinsic element for escape from XCI [9]. In this previous study, the authors inserted mice bacterial artificial chromosomes containing *Kdm5c*, which is an escape gene, with 112 kb surrounding sequences into the inactivated regions; however, *Kdm5c* remained an escape gene [9]. In humans, patients with X-autosome translocation showed inactivated autosomal genes by spreading XCI from Xi in *cis.* However, some autosomal genes within the region including inactivated autosomal genes escaped from the inactivation [10]. Furthermore, studies focusing on three-dimensional (3D) structure of X chromosomes have been reported. 3D structure is different between Xi and Xa [11]. Xa has topologically associating domains (TADs) which enhance intrachromosomal contacts, but Xi has attenuated TADs [11]. A macrosatellite repeat *DXZ4* is located at the boundary of the bipartite structure of megadomains on Xi [11, 12]. Xi has extremely long-range loops which occur among *XIST*/*Xist*, *DXZ4*/*Dxz4*, and *FIRRE*/*Firre*, and an inactive-X CTCF-binding contact element (ICCE) [13]. In mice, escape genes tend to be located in the peripheral regions of the 3D structure of Xi [12]. Disruption of 3D structure by deleting both *Dxz4* and *Firre* in mouse ES cells did not lead to disruption of XCI or change the total numbers of escape genes; however, it led to change in the contents of the escape genes list [11]. In humans, skewing XCI is observed in individuals with chromosomal abnormalities [14]; however, the effect of X-chromosome rearrangements on escape genes remains to be studied. Here, to reveal the impact on escape genes by X-chromosome rearrangements in humans, we examined the breakpoints and methylation status of the promoter regions in escape genes as well as in inactivated genes in patients with various X-chromosome rearrangements.

## Results

### Clinical features

Four 46,XX female patients with various X-chromosome rearrangements were included in this study. Patient 1 was previously reported [15]. She received genetic analysis due to abnormal menstruation. Patients 2 and 4 first visited the hospital to receive assessment for short stature. Patient 3 had severe intellectual developmental delay and epilepsy. All four patients did not exhibit Turner stigmata.

### Detecting of breakpoints in chromosomal abnormalities

**Fig. 1:**
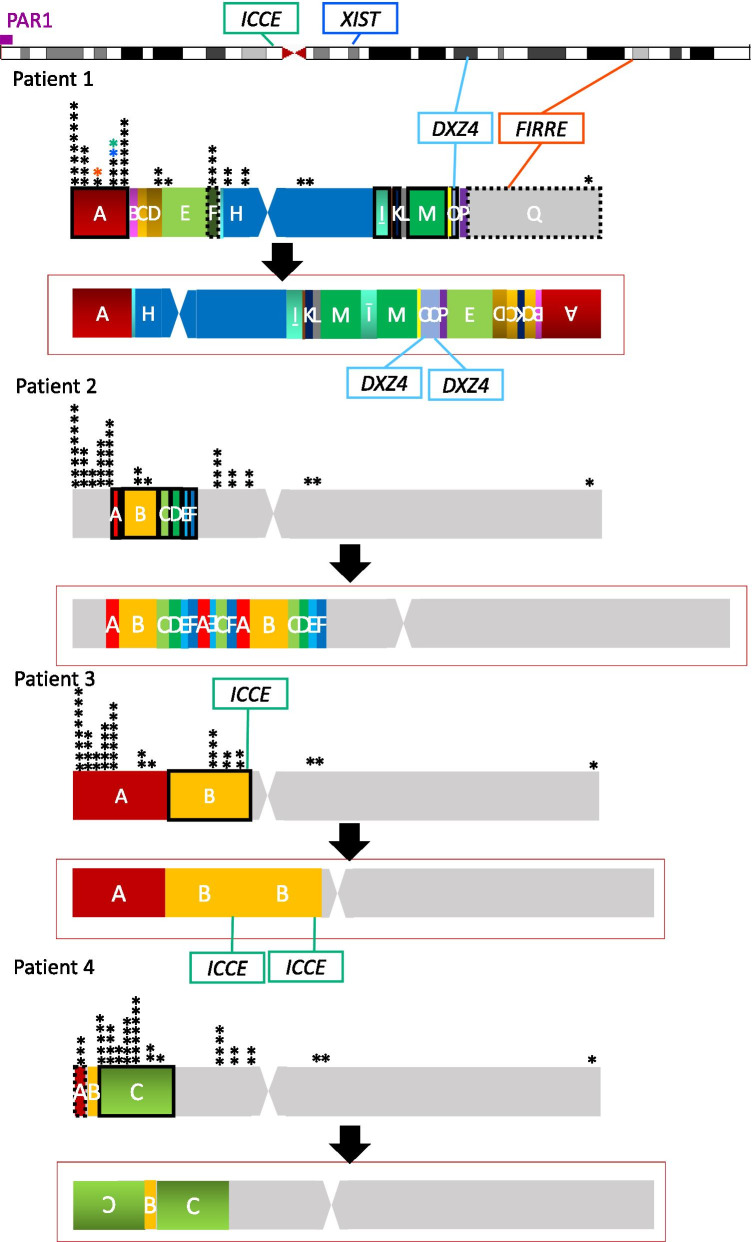
Rearrangements of the X chromosome in the four patients. The black down arrow exhibits the rearrangements of the X chromosome in each patient. The upper chromosome in each patient shows a normal X chromosome. A box surrounded with dashed lines means a deleted region. A box surrounded with bold lines means a duplicated region. The region with an upside-down alphabet had inversion. The locations of escape genes are shown as asterisks. The locations of the three genes with abnormal methylation in Patient 1 are exhibited using colored asterisks (red: *PNPLA4*, blue: *TCEANC*, green: *GPM6B*). The loci which are important for the 3D structure of Xi are also shown with surrounding squares in Patient 1 and Patient 3. When important loci for the 3D structure are not included in duplication or deletion in each case, we did not show them in the figure. No patients had *XIST* on rearrangements

Patient 1 had 46,X,der(X)(pter → p22.1::p11.23 → q24::q21.3 → q24::p11.4 → pter) karyotype detected by our previous study [15]. Karyotyping showed 46,X,add(X)(p21.1) in Patient 2, 46,X,dup(X)(p11.23p22.1) in Patient 3, and 46,X,add(X)(p22.32) in Patient 4. For detection of chromosomal breakpoints in the four patients with X-chromosome rearrangements, we conducted array-based comparative genomic hybridization (aCGH) and whole-genome sequencing (WGS) using genomic DNA (gDNA) samples from peripheral blood of the patients. After narrowing the possible ranges including breakpoints based on the results of aCGH (shown in Additional file 1: Figure S1), we estimated breakpoints based on the WGS data. Finally, we determined the breakpoints by direct sequencing of the PCR-amplified DNA fragments harboring the junction (Additional file 2: Table S1), although we could not confirm one or two of their fusion points in Patient 1 and Patient 2, respectively (Additional file 2: Table S1). Patient 1 had the most complicated rearrangements among the four patients. This rearranged X chromosome exhibited a 7 Mb deletion at Xp and a 36 Mb deletion at Xq, and totally 20 Mb and 25 Mb duplicated regions at Xp and at Xq, respectively (Fig. 1). Patient 1 had a duplication containing *DXZ4* and a deletion containing *FIRRE*. Patient 2 had a rearranged X chromosome with 11 Mb duplication and 0.8 Mb triplication at Xp (Fig. 1). As shown in Figure S1, the rearranged region on Xp had three or four copies. Patient 3 had a simple 29 Mb duplication at Xp containing *ICCE* (Fig. 1). Patient 4 exhibited a 0.7 Mb deletion at Xp including part of PAR1 and a duplication at Xp including an approximately 29 Mb inversion (Fig. 1). The copy number of *XIST* was not affected in any of the patients. Almost all the breakpoints in the patients were not located on CpG islands, high-density transcriptional factors or DNase clusters (Additional file 3: Figure S2).

### X-chromosome inactivation analysis

**Fig. 2:**
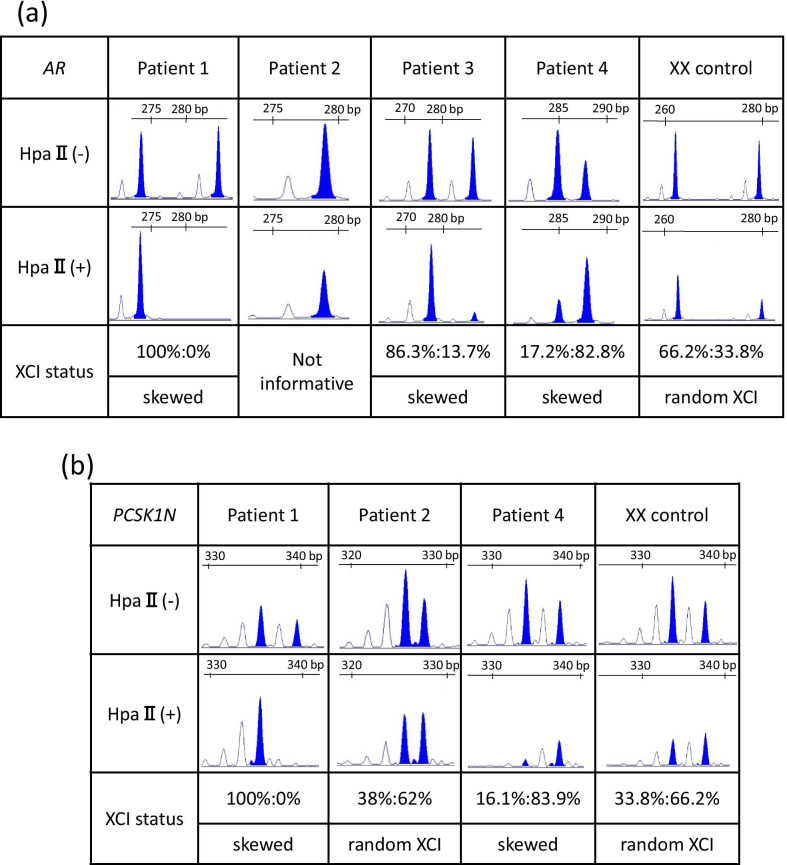
X-inactivation analysis for the *AR* and the *PCSK1N* genes. Microsatellite analysis of a polymorphic CAG repeat tract before and after digestion with HpaII in the *AR* gene (**a**) and the *PCSK1N* gene (**b**). XCI was regarded as being skewed when the ratio was more than 80% and as random XCI when the ratio was under 80% [17]

We conducted inactivation analysis and calculated the XCI ratio in the androgen receptor (AR) gene in all four patients and in the *PCSK1N* gene in all patients except for Patient 3 as previously reported [16, 17] (Fig. 2). An XCI ratio of 80% or more was regarded as skewed XCI based on criteria [17]. Inactivation analysis in the AR genes showed completely skewed XCI in Patient 1, and skewed XCI in Patients 3 and 4. Patient 2 showed an uninformative result due to only a single peak. Inactivation analysis in the *PCSK1N* gene showed random inactivation in Patient 2 (Fig. 2b).

### Array-based methylation analysis

**Fig. 3:**
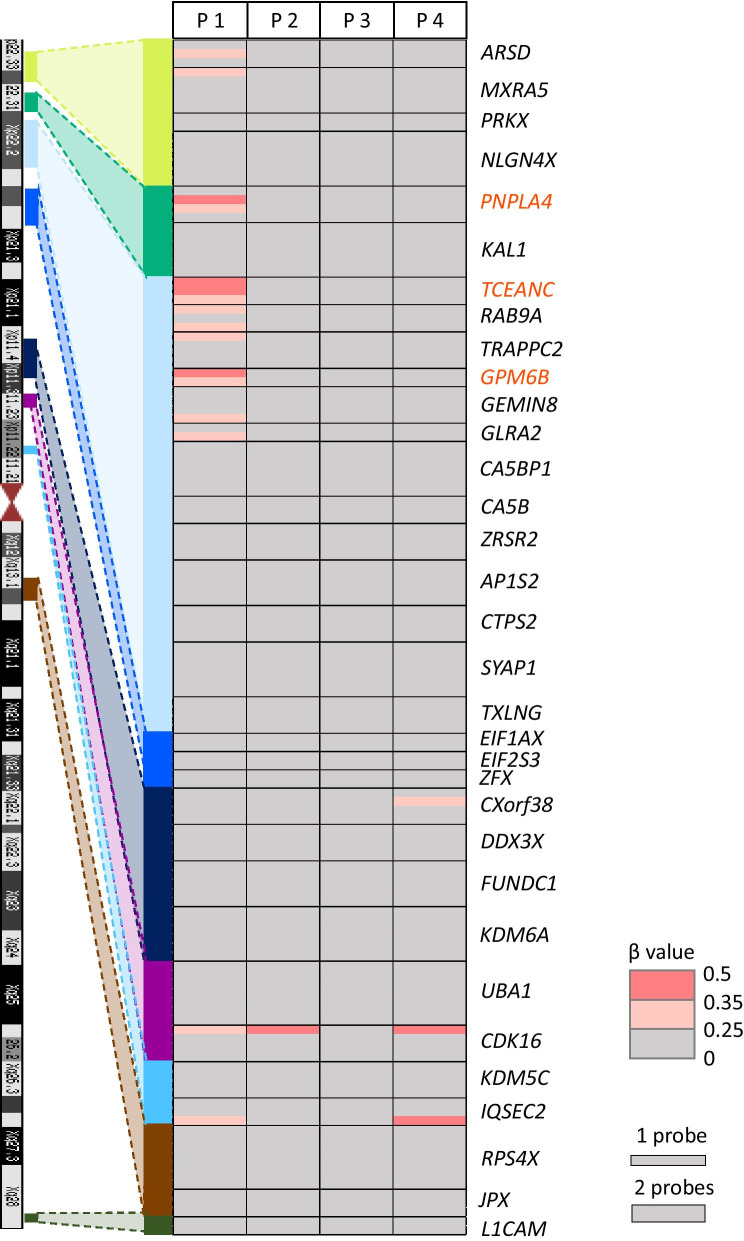
Methylation status at the promoter region of 32 escape genes examined by array-based methylation analysis using the Illumina Infinium Human Methylation EPIC BeadChip kit in the four patients. The heat map of *β* values in the four patients. The probes with lower than 0.25 in the *β* value are shown in gray and those with higher methylation (over 0.25) are shown in pink. The names of escape genes are shown at the right side of the heat map. The names of the three genes with abnormal methylation in Patient 1 are exhibited in red. The number of probes in each gene is shown as the height of each gene in the heat map (the height of one probe and two probes is exemplified at the lower right part of the figure)

To investigate the methylation status of the promoter regions of escape genes, we conducted array-based methylation analysis with gDNA from peripheral blood of four patients and 23 controls (four adult males, eight boys, four adult females, and seven girls) using Illumina Infinium Human Methylation EPIC BeadChip (Illumina Inc., San Diego, CA, USA), although we could not examine the methylation levels of the promoter regions of escape genes located in PAR1 due to no probes in EPIC. We examined the methylation levels of the promoter regions that had more than two consecutive hypomethylated probes (the mean *β* < 0.15) in both male and female controls and no large methylation difference between both sexes (|Δ*β*|< 0.1) within 1 kb from transcription start sites (TSS). We extracted 44 genes with a hypomethylated promoter region. Thirty-two of these genes (72.7%) were identical to escape genes or mostly escape genes in a previous report (Additional file 4: Figure S3 and Additional file 5: Table S2) [18]. The previous report by Balaton, et al. aggregated three published studies on inactivated status of genes on the X chromosome by gene expression analysis using hybrid cells, the expression imbalance of X-linked SNPs between the allele on Xa and Xi, and by comparing the methylation level at the promoters of genes between females and males [18]. In this report, when a gene was regarded as an escape gene in all three studies, it was called an escape gene, and when a gene was regarded as an escape gene in the majority of the studies, it was categorized as a mostly escape gene [18]. We regarded 32 genes as escape genes and evaluated the methylation levels of the probes included in the promoter regions of these 32 genes (Additional file 5: Table S2) in four patients. In Patient 1, 18 escape genes and 5 escape genes were on the duplicated region and deleted region, respectively. Three genes in Patient 2, 8 genes in Patient 3, and 21 genes in Patient 4 were on their duplicated regions (Additional file 5: Table S2). We extracted genes of which the promoter had more than two consecutive probes with abnormally high methylation levels (*β* value > 0.25). Patients 2, 3, and 4 did not have genes with abnormally hypermethylated promoter regions in those escape genes. Patient 1 had three genes with abnormally hypermethylated promoter regions, *PNPLA4*, *TCEANC*, and *GPM6B* (Fig. 3, Additional file 6: Table S3). These three genes were on a duplicated region located from 5.5 to 10 Mb away from the breakpoint at Xp (Fig. 1). Although the *CDK16* gene at proximal Xp showed a hypermethylated probe in three patients, more than two consecutive hypermethylated probes in this gene were not identified (Fig. 3, Additional file 6: Table S3).

We also examined the methylation levels of the promoter regions in inactivated genes. We extracted 137 genes which had more than two consecutive probes hypomethylated in male controls (the mean *β* value < 0.15 and all male controls’ *β* value < 0.25) and not hypomethylated in female controls (all female controls’ *β* value > 0.25 and the mean *β* value < 0.5) within 1 kb from TSS. One hundred and twenty-five of these genes were identical to subject genes or mostly subject genes in the previous report [18]. We regarded those 125 genes as inactivated genes and evaluated the methylation levels of the probes included in the promoter regions of these genes in four patients (Additional file 7: Table S4). We extracted genes of which the promoter had more than two consecutive probes with hypomethylated levels (the *β* value < 0.15) from those inactivated genes. Patients 2, 3, and 4 had no inactivated genes with hypomethylated promoter regions (Additional file 7: Table S4). Patient 1 had 38 inactivated genes with the hypomethylated promoter regions, and all the genes were on the deleted regions (Additional file 7: Table S4). Furthermore, we extracted the genes of which the promoter had more than two consecutive probes with hypermethylated levels (*β* value > 0.5 and *β* value > the max *β* value in female controls + 0.05). Patient 1 had two inactivated genes with hypermethylated promoter regions on the duplicated regions (Additional file 7: Table S4), and Patients 2–4 had no inactivated genes with hypermethylated promoter regions, even on duplicated regions (Additional file 7: Table S4).

### Reduced representation bisulfite sequencing (RRBS)

To examine the methylation status of the promoter regions of escape genes located in PAR1, we conducted RRBS in the four patients and eight controls (four males and four females) and evaluated the methylation levels of cytosine-phosphate-guanine (CpG) sites in the promoter region within 1 kb from TSS. Eight genes with hypomethylated promoter regions were detected based on our criteria as follows: (1) the gene had more than two CpG sites exhibiting hypomethylation (mean methylation ratio < 0.15) in both male controls and female controls in the promoter region; (2) CpG sites did not show large methylation differences between males and females (difference in methylation ratio in the mean of controls between both sexes ranged from − 0.1 to 0.1); (3) CpG sites with a methylation ratio > 0.24 in one or more controls were excluded from analysis. We evaluated the methylation ratio of CpG sites at the promoter regions in these eight escape genes (Additional file 5: Table S2). Eight escape genes were on the duplicated region in Patient 1, and three and five escape genes were on the deleted region and duplicated region, respectively, in Patient 4. We found no patients showing abnormal hypermethylation (methylation ratio > 0.25) in more than two CpG sites at the promoter region of those escape genes (Additional file 8: Table S5).

## Discussion

The X-chromosome rearrangements in our patients did not affect the methylation status of the promoter region in most escape genes. Patients 2–4 showed no methylation abnormality in the promoter regions of escape genes, and Patient 1 had abnormally hypermethylated promoter regions in only three escape genes on the duplicated region. These results suggest that rearrangements detected in these patients might not include the critical regions for the regulation of the methylation status of almost all escape genes. Escape genes (except for three) did not have methylation abnormality and seemed to successfully escape from XCI, even though some genes were located at a different region from the original region due to the rearrangements.

Only three genes included in the duplicated region showed abnormal hypermethylation in Patient 1 with the most complex X-chromosome rearrangements. The methylation abnormalities in the three genes in Patient 1 may be attributed to the highly complex rearrangements and/or disrupted 3D structure of Xi. Because the promoters of the other escape genes involved in the same duplicated regions remained to be hypomethylated, it is unlikely that duplication itself caused abnormal promoter methylation in these three genes. Patient 4 also had a larger duplication containing these three genes, but this patient did not show methylation abnormality in these three genes. In both patients, these three genes were more than 5 Mb away from the nearest breakpoints; however, the methylation status of the genes was different between Patients 1 and 4. These results suggest that surrounding sequences of these three genes within 5 Mb might not be responsible for escape from XCI. Patient 1 had much more complicated rearrangements compared to those of Patient 4, and the duplicated regions containing these three genes were transferred from the original location at Xp to Xq. The promoter of these three genes may be vulnerable to the inactivated status in the surrounding region on Xq. We also focused on a deletion of the *FIRRE* gene and a duplication of *DXZ4* in Patient 1. Both *DXZ4* and *FIRRE* are in the important loci forming the 3D structure of Xi [11, 13]. Deleting *Dxz4* in mice showed impairment of the formation of the megadomain boundary [11, 13], and deletion of the *Firre* gene in mice showed disruption of the superloop between *Dxz4* and *Firre* and attenuates intra-megadomain interactions [11, 13]. Deletion of *Firre* and *Dxz4* in mouse ES cells led to disruption of the 3D structure of Xi, but not impairment of expression of escape genes and the total number of escape genes; however, the contents of escape genes partially changed [11]. In humans, it was reported that a case with a 32 Mb deletion containing *FIRRE* on Xq showed upregulation in one escape gene and some inactive genes and downregulation in variable escape genes on a normal copy number region [19]. This case also showed compensatory upregulation of escape genes on a deleted region [19]. On the other hand, in Patient 1 with the deletion of *FIRRE* and the duplication of *DXZ4,* three escape genes showed hypermethylation. Further studies combining methylation analysis with expression analysis are needed to clarify the role of the *FIRRE/Firre* gene for escape genes. It was reported that *ICCE* also loop with *DXZ4* and *FIRRE* only in higher primates [20]. Patient 3 had a duplication of *ICCE* and did not exhibit methylation abnormalities in the promoter region of escape genes. The rearrangements containing *ICCE* may also disrupt the 3D structure of Xi, but the impact of duplication of *ICCE* on the 3D structure is unclear.

For inactivated genes, Patient 1 had 39 genes with the hypomethylated promoter regions on the deleted regions and two genes with obviously hypermethylated promoter regions on the duplicated regions. Patients 2–4 had no genes with the hypermethylated promoter regions on the duplicated regions. A previous report showed that skewed XCI is observed in individuals with chromosomal abnormalities [14]. However, to our knowledge, the methylation status of the promoter region of inactivated genes on the duplicated region in the patients with structural abnormalities of the X chromosome has not been studied. In a previous report, two female patients showing moderate mental retardation, who had *MECP2* duplication with random XCI, were discussed [21]. Because *MECP2* on the X chromosome is the inactivated gene, female duplicated cases with skewed XCI are asymptomatic [21]. Clinical symptoms in these reported cases may be caused by increased *MECP2* gene expression due to random XCI [21]. These findings suggest that duplicated regions on Xi are likely to be inactivated. In our study, the levels of the skew of XCI are various. Patient 1 had hypomethylated promoters of the inactivated genes on the deleted region, and obvious hypermethylated promoters of two inactivated genes on the duplicated genes. On the other hand, Patients 2–4 had no obvious hypermethylated promoters of inactivated genes on the duplicated genes. These findings suggest that the abnormal hypermethylation of inactivated genes in these three patients might be underestimated. In cases with incompletely skewed XCI, inactivated genes on the duplicated region are not fully inactivated because some X chromosomes with the duplication are Xa. Therefore, we speculate that the differences in the status of the skewed XCI affect the methylation levels of the promoter regions of inactivated genes on the duplicated region. In addition, it is still unclear whether the duplicated regions on Xi are fully inactivated. Further study is necessary to reveal this.

There are some limitations of this study. First, because our study is only based on the results of methylation analysis for the promoter regions of the escape genes, we could not evaluate the expression of the genes regarded as failing to escape from XCI in Patient 1. We consider that methylation analysis could be useful to estimate the escape from XCI according to previous reports examining the status of XCI and escape by methylation analysis [4, 10]. Second, because we obtained only the genomic DNA sample of Patient 1, we could not perform 3D structural analysis. Therefore, we could not assess the correlation among methylation status, 3D structure, and gene expression on the X chromosome. Third, the differences in the status of XCI could cause the differences in the detection rate of the change in the methylation levels in the escape genes. In brief, Patient 1 showed completely skewed XCI. On the other hand, Patients 2–4 showed partially skewed XCI or random XCI. Because an X chromosome with structural abnormality is inactivated [14], all escape genes with aberrant hypermethylation in Patient 1 are on Xi. In Patients 2–4, some X chromosomes with structural abnormality are Xa. Thus, the methylation change in escape genes on an X chromosome with rearrangements would be masked and less detectable in Patients 2–4 compared to Patient 1. In fact, inactivated genes on duplicated regions in Patients 2–4 did not show hypermethylation.

In conclusion, X-chromosome rearrangements are unlikely to affect the methylation status of the promoter regions of escape genes, except for a specific case with complex rearrangements.

## Methods

### Subjects

Four female patients with X-chromosome rearrangements were included in this study. Twenty-three controls (four adult males, eight boys, four adult females and seven girls) were included in array-based methylation analysis and another eight controls (four males and four females) were included in RRBS analysis.

### Sample preparation

Genomic DNA (gDNA) of four patients and 31 controls (23 controls for EPIC and 8 controls for RRBS) was extracted from leukocytes of peripheral blood using a commercial DNA extraction kit.

### Breakpoint detection

To detect breakpoints, we first performed aCGH analysis using a catalog array (catalog number G4448A or G4449A, Agilent Technologies, Palo Alto, CA, USA) using gDNA of four patients with X-chromosome rearrangements. Next, we carried out WGS. Genomic libraries were constructed with the Illumina TruSeq DNA PCR-Free kit and sequenced using the Hiseq X-ten sequencer (Illumina) with 150-bp pair-end reads. The sequences of library adaptors were removed by using cutadapt 2.6. We mapped sequence reads against the hg19/GRCh37 reference sequence using the Burrows-Wheeler Aligner (BWA) 0.7.15. PCR duplicates were removed by Picard 2.17.11. After local realignment of the X chromosome using GATK 3.8, we estimated the position of the junction in each patient on the Integrative Genomics Viewer (IGV) with BAM file data. Finally, we identified the genomic position of each breakpoint by direct sequencing of the PCR-amplified DNA fragments harboring the junction.

### Inactivation analysis

To evaluate the X-inactivation status, we performed inactivation analysis as previously reported [16, 17]. In brief, we performed PCR amplification for CAG repeats in exon 1 of the *AR* gene [16] and those in intron 1 of the *PCSK1N  *gene [17] using gDNA of the patients and female controls treated with or without HpaII, which is a methylation-sensitive restriction enzyme. Subsequently, we subjected PCR products on the ABI 3130xl and 3500xl auto-sequencer and evaluated the heights of peaks of PCR products using GeneScan software (Applied Biosystems, Foster City, CA, USA). The XCI ratio was calculated by dividing the heights of peaks of PCR products using gDNA treated with HpaII by those of PCR products using gDNA treated without HpaII. We performed inactivation analysis for the *AR* gene in four patients and one normal female control [16] and for the *PCSK1N* gene in Patients 1, 2, and 4, and one normal female control. Because the duplicated region of Patient 3 included the *PCSK1N* gene, we excluded this patient in the assay of *PCSK1N*. XCI was regarded as being skewed when the ratio was more than 80% [17] and as random XCI when the ratio was under 80%.

### Array-based methylation analysis

gDNA samples from the patients and controls were treated with bisulfite using the EpiTect plus DNA bisulfite kit (Qiagen, Hilden, Germany). We subjected bisulfite-treated gDNA to the Illumina Infinium Human Methylation EPIC BeadChip (Illumina) and scanned by the Illumina iScan system. All statistical tests were conducted by R version 3.4.1. We used the R package called Chip Analysis Methylation Pipeline (ChAMP) version 2.12.3 [22], Beta MIxture Quantile dilation (BMIQ) [23], and ComBat for the bioinformatics analysis [24]. First, for data preprocessing, raw IDAT files were imported with the ‘champ.load’ function in ChAMP with all the default conditions, except for removing the filter for sex chromosomes and changing the array type to EPIC. We removed the probes with less than three beads, probes containing SNPs, and probes with detection *P* values above 0.01. Next, the BMIQ methods were utilized for quantile normalization. Subsequently, we used ComBat to correct for the batch effect [24]. Lastly, to detect the probes with age-related drift, we exploited the ‘champ.DMP’ function in ChAMP for finding probes with significant differentially methylated levels between adult female controls and girl controls, or between adult male controls and boy controls. After excluding probes with the adjusted *P* value of < 0.05 between adult controls and child controls, we extracted 7485 probes from the X chromosome. The DNA methylation level at each probe was converted to *β* values ranging from zero to one, which meant completely unmethylated and completely methylated, respectively.

We determined the loci within 1 kb from TSS as the promoter region of the escape gene according to a previous study [4]. When more than two consecutive probes in the promoter region of the gene satisfied the following criteria in controls, namely (1) with hypomethylation (the mean *β* value was less than 0.15 and *β* values in all controls were below 0.24) in both sexes, and (2) without a large methylation difference between males and females (|Δ*β*|< 0.1), we extracted the gene. Forty-four genes were extracted, and 32 of them were identical to escape genes which were evaluated by gene expression analysis using hybrid cells in the previous report [18]. We evaluated these 32 genes in our patients. To clarify whether X-chromosome rearrangements affect the methylation levels of the promoter region of escape genes, we examined the methylation levels and extracted the escape genes with more than two consecutive probes having a higher methylation level (*β* value > 0.25) in the promoter region. To extract inactivated genes, we examined the methylation levels of the promoter regions that had more than two consecutive probes which were hypomethylated in male controls (the mean *β* value was less than 0.15 and *β* values in all male controls were below 0.25) and not hypomethylated in female controls (*β* values in all female controls were above 0.25 and the mean *β* value was less than 0.5) within 1 kb from TSS. According to these criteria, we extracted 137 genes. One hundred and twenty-five of these genes were identical to subject genes or mostly subject genes in the previous report [18]. We regarded those 125 genes as inactivated genes, and evaluated the methylation levels of the probes in the promoter regions of these 125 genes in four patients. We extracted genes of which the promoter had more than two consecutive probes with hypomethylated levels (*β* value < 0.15). Furthermore, we extracted genes of which the promoter had more than two consecutive probes with hypermethylated levels (*β* value > 0.5 and *β* value > the max *β* value in female controls + 0.05).

### RRBS

To evaluate the methylation status of escape genes in PAR1, we conducted RRBS with gDNA of four patients and eight controls (four males and four females) according to a previous report [25]. Briefly, 300 ng gDNA samples were sheared using the S220 Focused-Ultrasonicator (Covaris Inc., Woburn, MA, USA) for the RRBS library. The sheared gDNA samples were treated with RNase and protease K and digested with MspI. Gap filling and A-tailing for sheared gDNA samples used the Klenow Fragment (Thermo Fisher Scientific, Waltham, MA, USA) and dNTP mixture (Qiagen). The A-tailed DNA was ligated with methylated adaptor (NEB Next Multiplex Oligos for Illumina, New England BioLabs, Ipswich, MA, USA) and subsequently bisulfite converted with the EZ Methylation Gold Kit (Zymo Research, Irvine, CA, USA) and amplified by PCR. The PCR products were size-selected and cleaned-up using Agencourt AMPure XP beads (Beckman Coulter Inc., Brea, CA, USA). The libraries of patients were sequenced using the Illumina NextSeq (Illumina) platform. The libraries of controls were sequenced using the Illumina HiSeq X Ten (Illumina) platform. The sequencing reads were mapped to the human reference genome (hg19/GRCh37) using the Bismark program [26]. We used Trim Galore software (http://www.bioinformatics.babraham.ac.uk/projects/trim_galore/) for adapter trimming and quality control and utilized the methylKit package version 1.12.0 [27] to count the reads of C or T at each CpG site and integrate the counted data of all samples. The counted data was annotated using HOMER software version 4.11 [28]. We excluded CpGs that had less than 10 read depth in one or more samples from data analysis. The methylation ratio from zero (completely unmethylated) to one (completely methylated) was calculated by dividing the read count of C by the total read count.

We extracted the genes in PAR1 of which the promoter regions within 1 kb from TSS had more than two CpGs satisfying the following criteria in controls, namely (1) with hypomethylation (mean methylation ratio < 0.15) in both sexes, and (2) without a large methylation difference between the mean of males and that of females (difference ranged from − 0.1 to 0.1). Of CpGs in the promoter regions of the extracted genes, we excluded CpGs in which one or more of the controls did not show hypomethylation (methylation ratio > 0.24) and CpGs exhibiting individual differences among controls. Finally, eight genes in PAR1 satisfied the criteria. We examined the methylation levels of the promoter regions of these eight genes in the four patients, and extracted genes which had more than two CpGs with the abnormally higher methylation levels (methylation ratio > 0.25).

## Supplementary Information

**Additional file 1: Figure S1.** Array-based comparative genomic hybridization of X chromosome of the patients.

**Additional file 2: Table S1.** Confirmed or estimated breakpoints in four patients.

**Additional file 3: Figure S2.** Breakpoint location.

**Additional file 4: Figure S3.** The number of escape genes.

**Additional file 5: Table S2.** The copy number of each escape gene in four patients.

**Additional file 6: Table S3.** The *β* value in promoter regions of escape genes in the patients.

**Additional file 7: Table S4.** The *β* value in promoter regions of inactivated genes in the patients.

**Additional file 8: Table S5.** The methylation ratio of CpGs in promoter regions of genes in PAR1.

## Data Availability

All data generated or analyzed during this study are available from the corresponding author on reasonable request.

## Abbreviations

aCGH: Array-based comparative genomic hybridization
AR: Androgen receptor
BMIQ: Beta MIxture Quantile dilation
BWA: Burrows-Wheeler Aligner
ChAMP: Chip Analysis Methylation Pipeline
CpG: Cytosine-phosphate-guanine sites
gDNA: Genomic DNA
IGV: Integrative Genomics Viewer
PAR1: Pseudoautosomal region 1
RRBS: Reduced representation bisulfite sequencing
TADs: Topologically associating domains
TSS: Transcription start sites
WGS: Whole-genome sequencing
Xa: Activated X chromosome
XCI: X-chromosome inactivation
Xi: Inactivated X chromosome
3D: Three-dimensional
