# Supplementary material for: Methylation status of genes escaping from X-chromosome inactivation in patients with X-chromosome rearrangements
Source: Clin Epigenetics. 2021 Jun 30;13:134. doi: 10.1186/s13148-021-01121-6 (PMC8244138; doi:10.1186/s13148-021-01121-6)
Supplement: Supplementary file 1 — Additional file 1: Figure S1. Array-based comparative genomic hybridization of X chromosome of the patients. [file 13148_2021_1121_MOESM1_ESM.pdf]

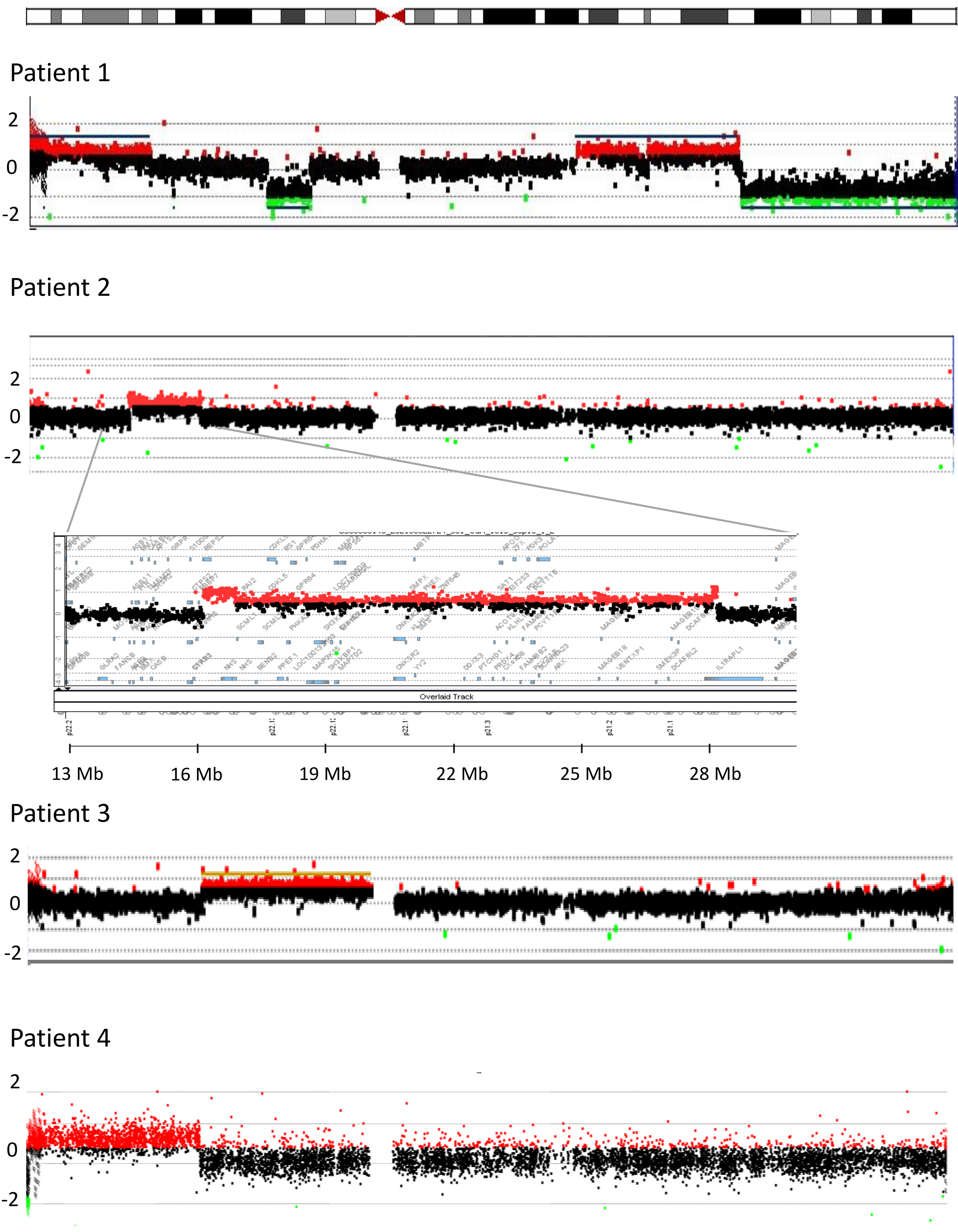

Figure S1. Array-based comparative genomic hybridization of X chromosome of the patients. The black dots show normal copy numbers. The red dots denote increased (log ratio higher than +0.4) copy numbers. The green dots show decreased (log ratio lower than –0.8) copy numbers. Patient 2's Xp was partially enlarged in order to show the copy number could be partially four.
