# Supplementary material for: Methylation status of genes escaping from X-chromosome inactivation in patients with X-chromosome rearrangements
Source: Clin Epigenetics. 2021 Jun 30;13:134. doi: 10.1186/s13148-021-01121-6 (PMC8244138; doi:10.1186/s13148-021-01121-6)
Supplement: Supplementary file 2 — Additional file 2: Table S1. Confirmed or estimated breakpoints in four patients. [file 13148_2021_1121_MOESM2_ESM.pdf]

Table S1. Confirmed or estimated breakpoints in four patients

Patient	Fusion junction	Chromosomal position (hg19)	Confirmed by direct sequencing or estimated
1	#1	18,222,188	confirmed
		46,828,523	confirmed
	#2	46,810,797	estimated
		46,830,605	estimated
	#3	114633605	confirmed
		99,658,792	confirmed
	#4	91,203,064	confirmed
		103,226,951	confirmed
	#5	118,305,930	confirmed
		114,665,469	confirmed
	#6	118,824,468	confirmed
		24,083,844	confirmed
	#7	39,669,564	confirmed
		23,983,985	confirmed
	#8	18,418,446	confirmed
		101,390,919	confirmed
	#9	99,818,115	confirmed
		20,124,322	confirmed
2	#1	29,351,253	estimated
		16,986,529	estimated
	#2	17,729,202	confirmed
		29,348,154	confirmed
	#3	29,348,219	confirmed
		28,793,876	confirmed
	#4	28,904,061	confirmed
		29,348,217	confirmed
	#1'	29,351,253	estimated
		16,986,529	estimated
3	#1	58,430,821	confirmed
		29,672,869	confirmed
4	#1	760,749	confirmed
		758,769	confirmed
