# Supplementary figures and images for: Methylation status of genes escaping from X-chromosome inactivation in patients with X-chromosome rearrangements
Source: Clin Epigenetics. 2021 Jun 30;13:134. doi: 10.1186/s13148-021-01121-6 (PMC8244138; doi:10.1186/s13148-021-01121-6)

Patient 1

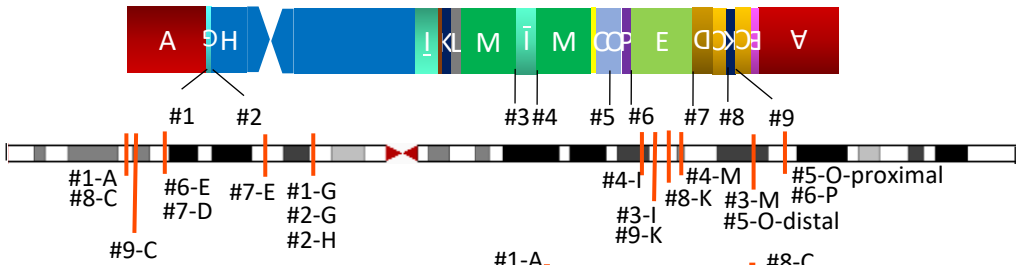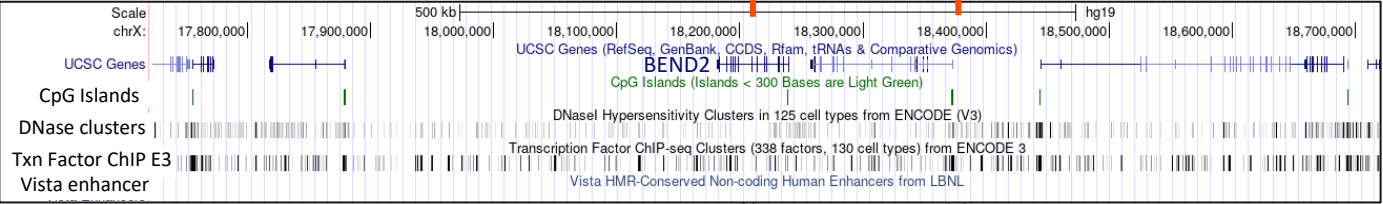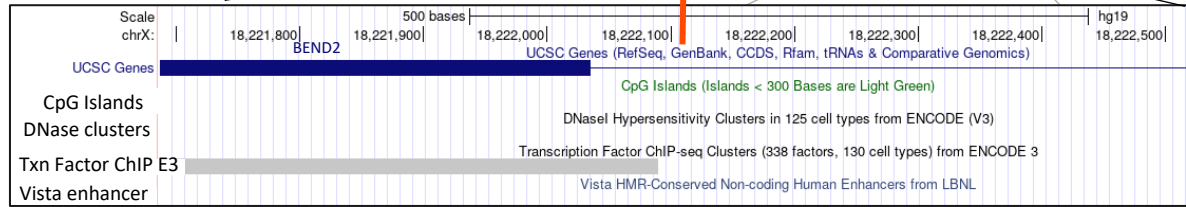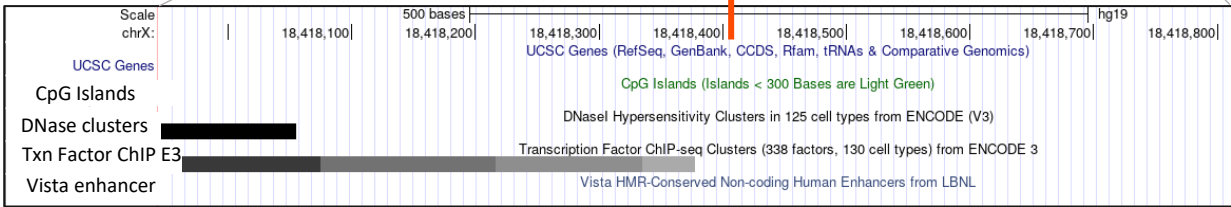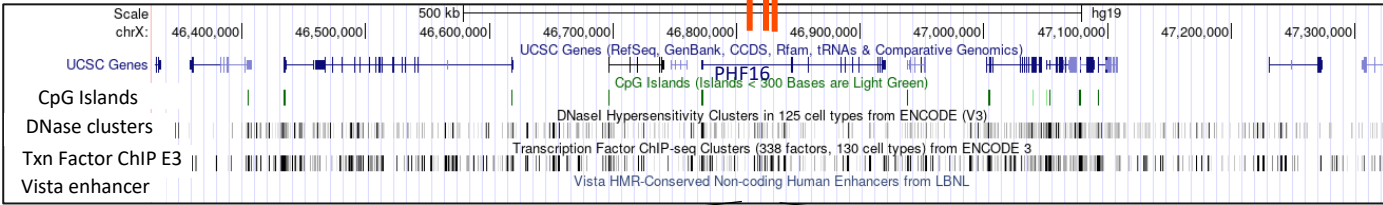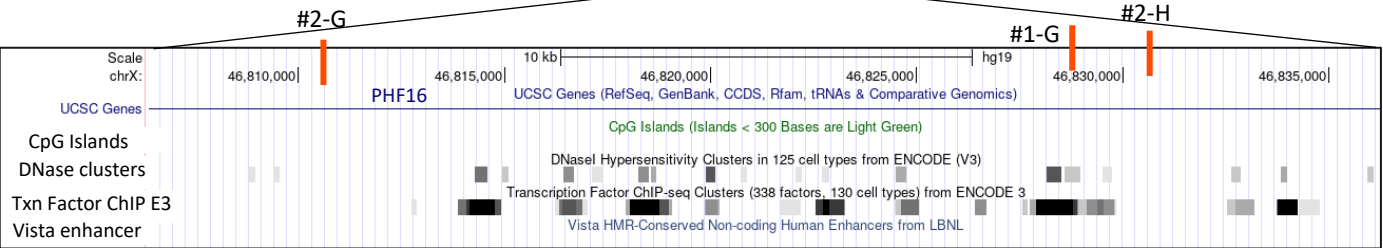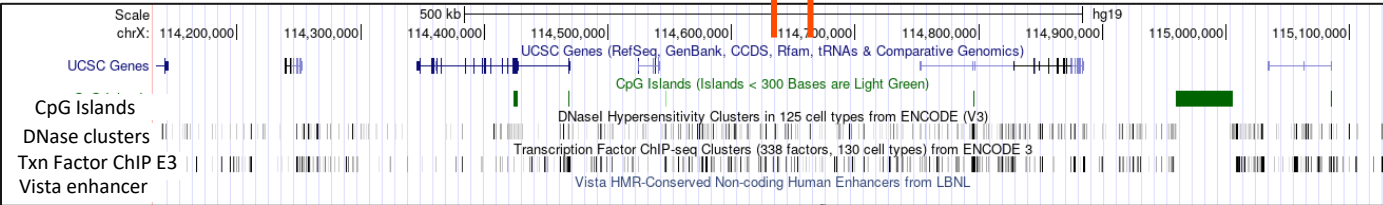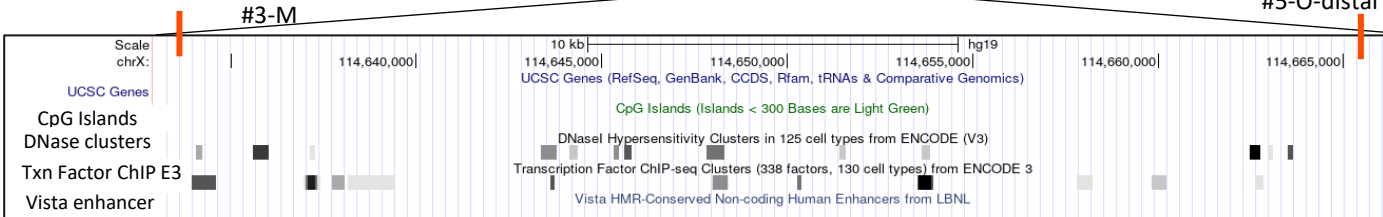

Patient 1

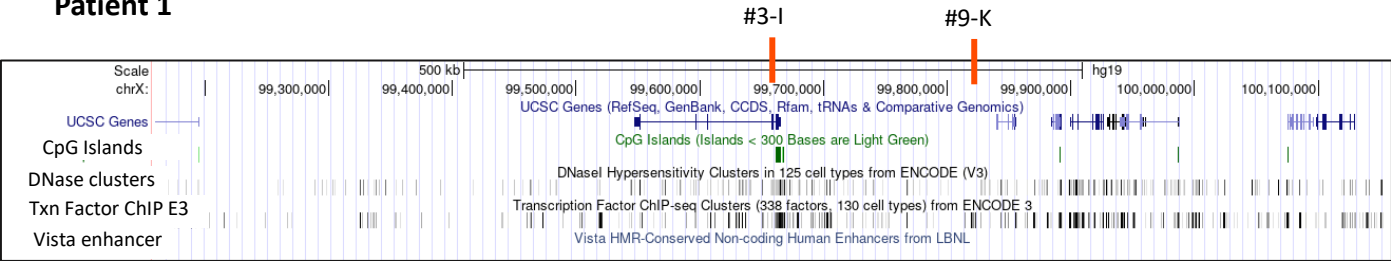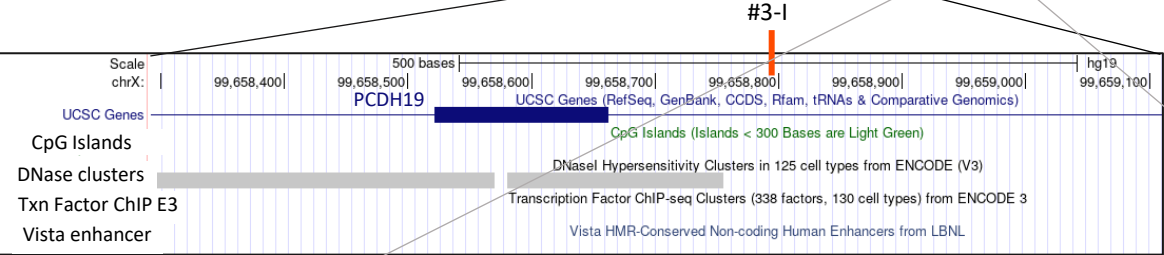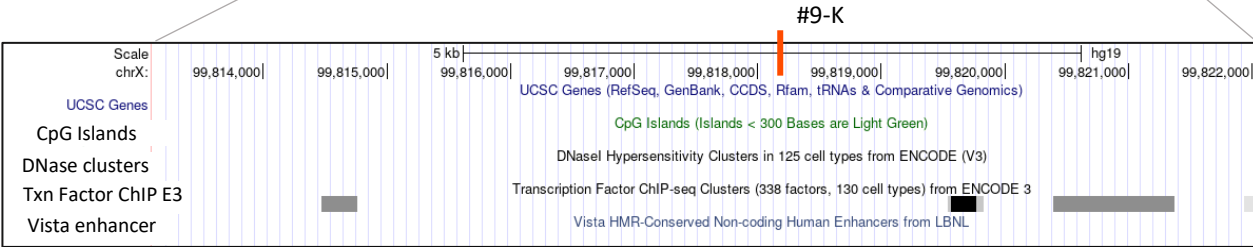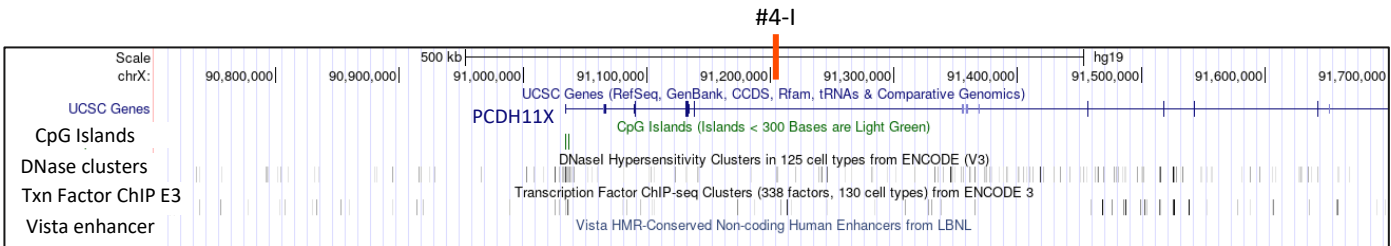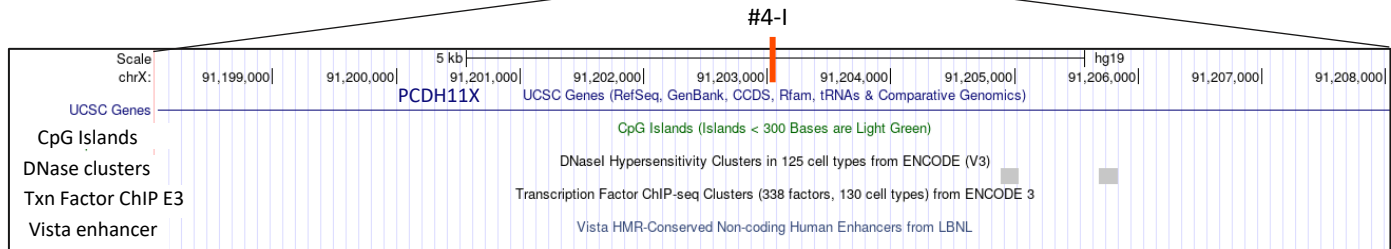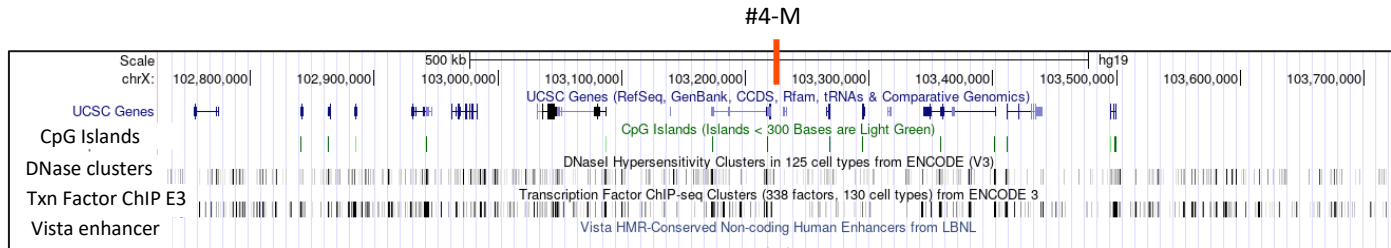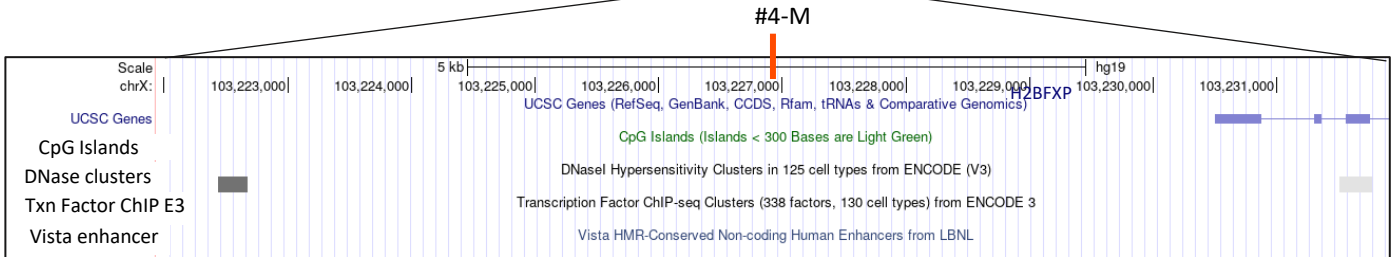

Patient 1

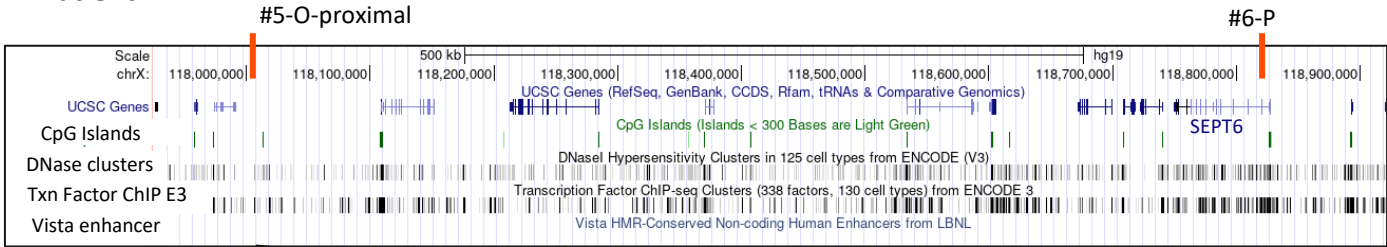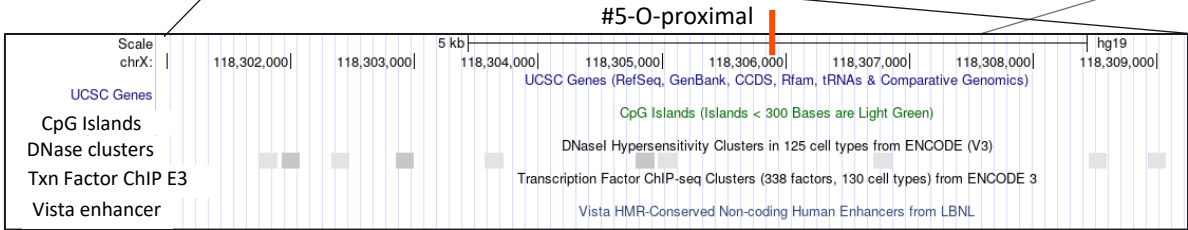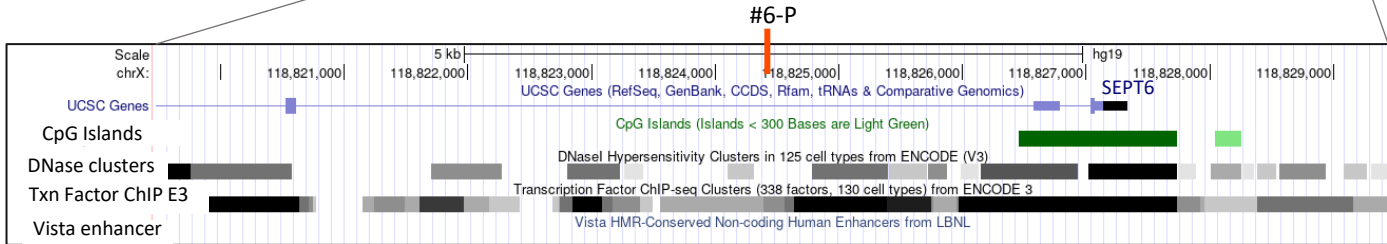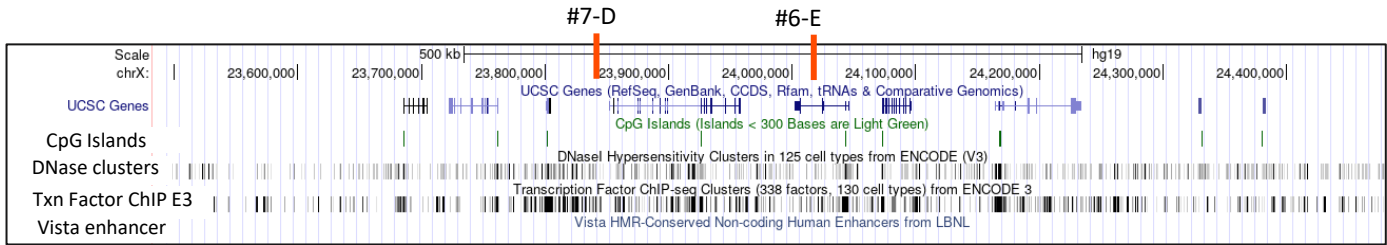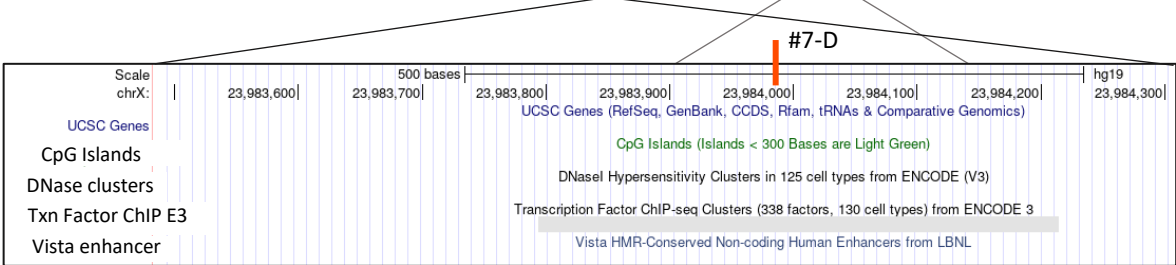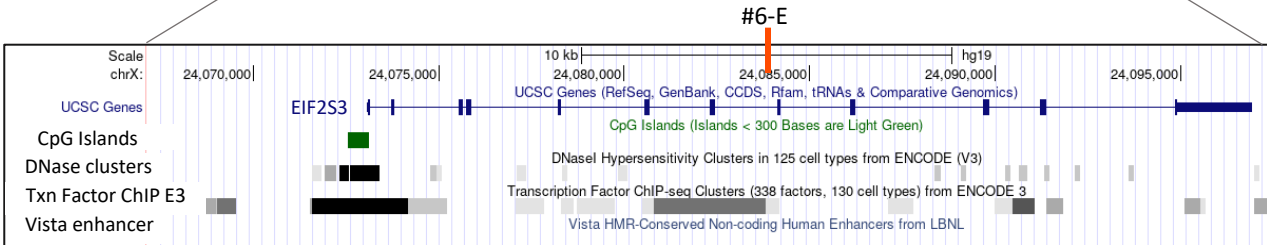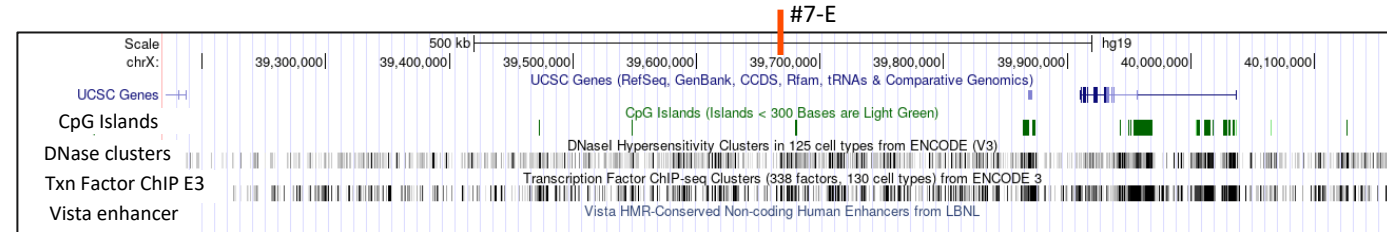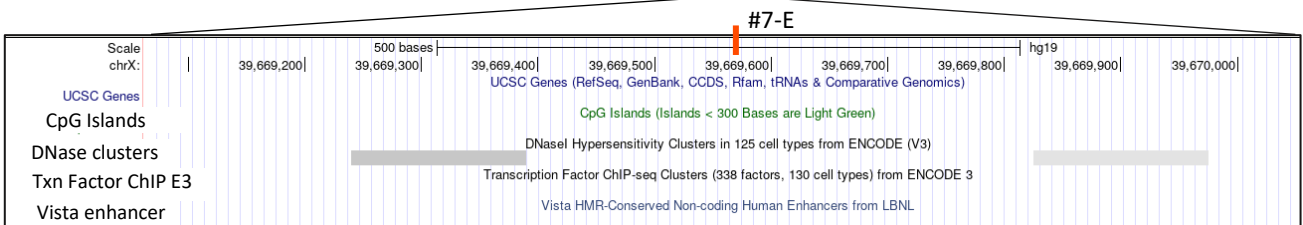

Patient 1

#8-K

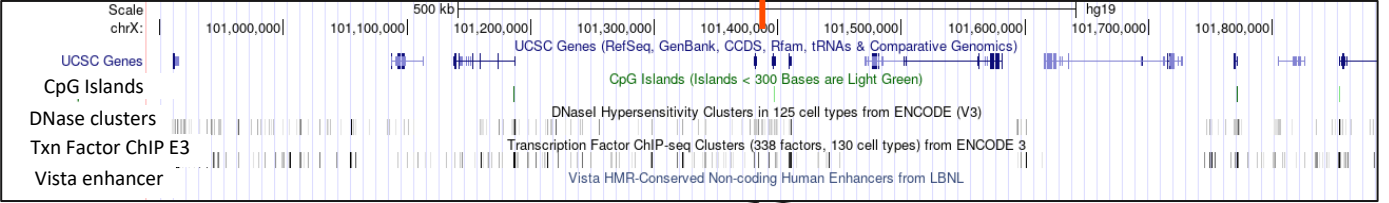

#8-K

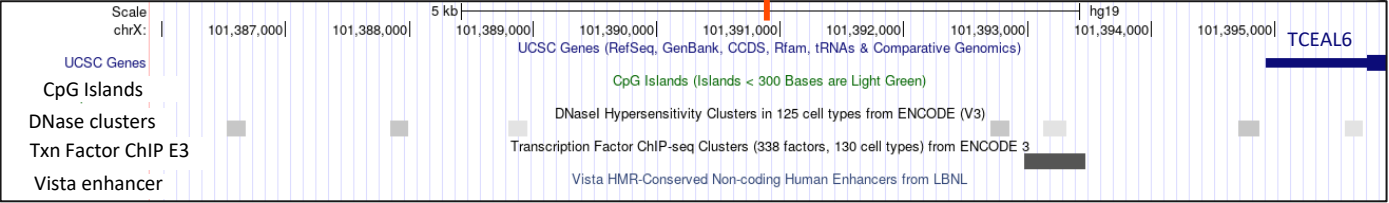

#9-C

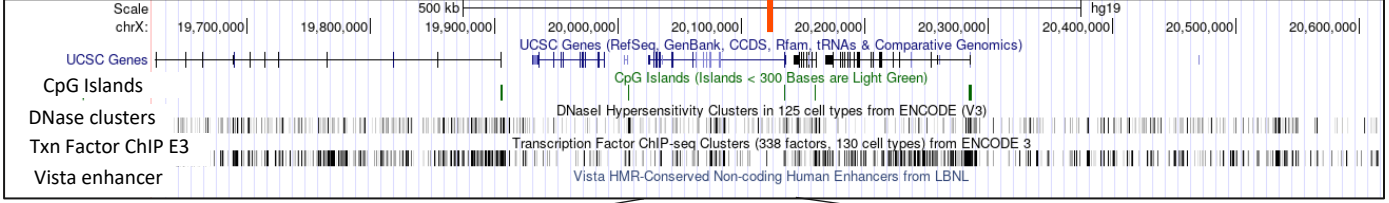

#9-C

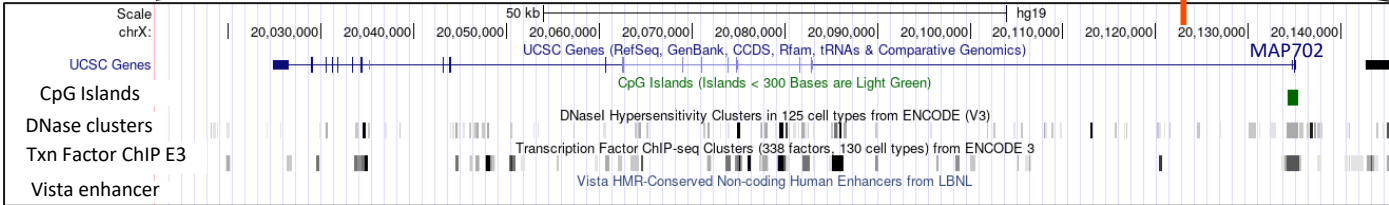

Patient 2

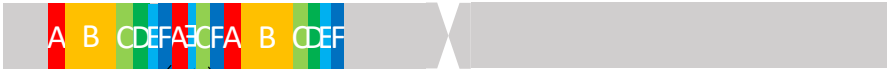

#1  
#2  
#3  
#4

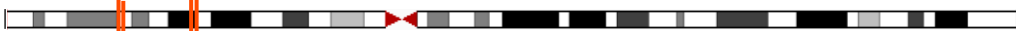

#1-A  
#2-A  
#3-C  
#4-C  
#1-F  
#2-E  
#3-E  
#4-F  
#1-F

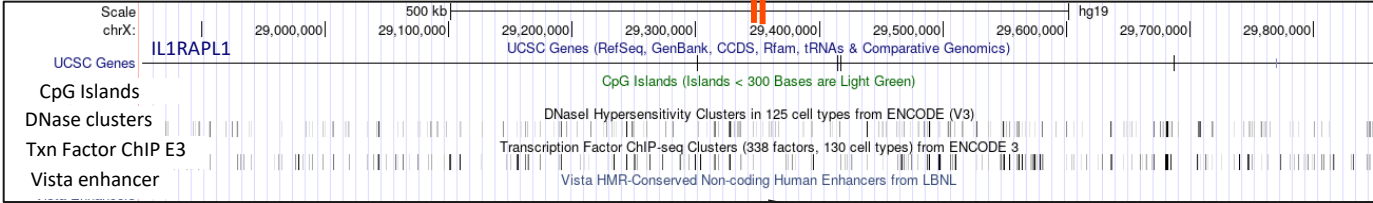

#3-E  
#2-E  
#4-F  
#1-F

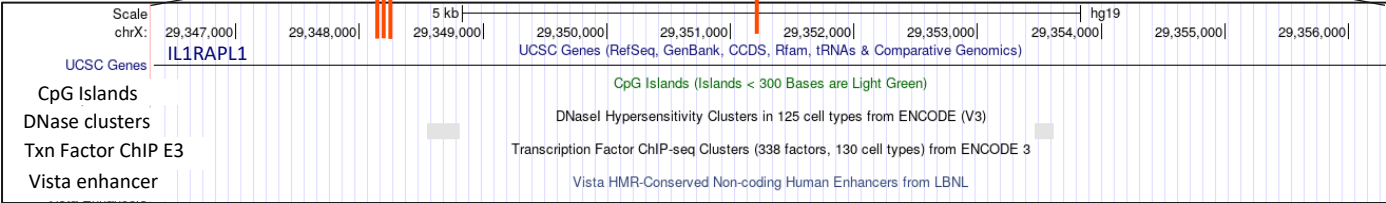

#1-A

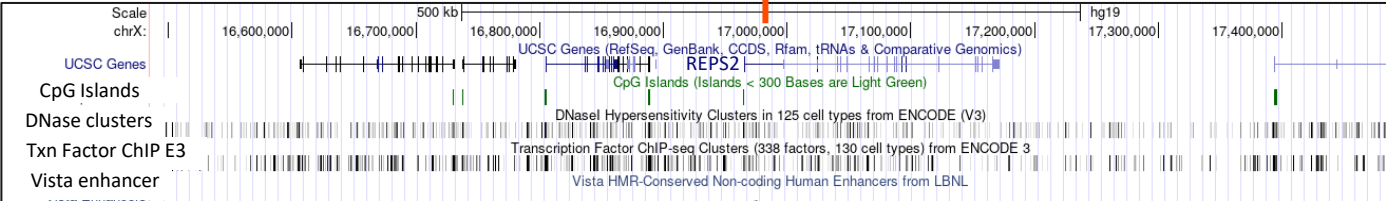

#1-A

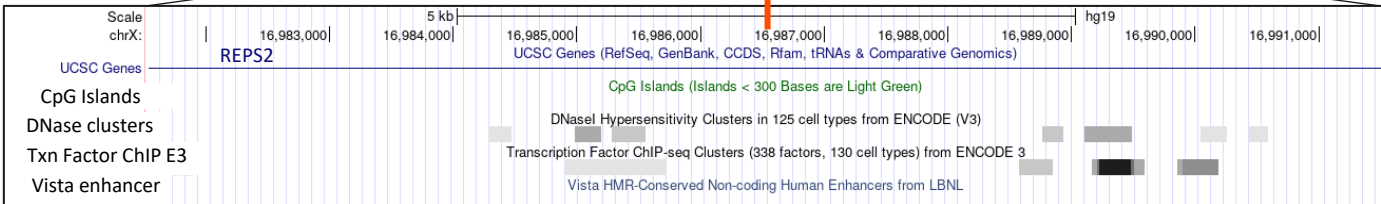

Patient 2

#2-A

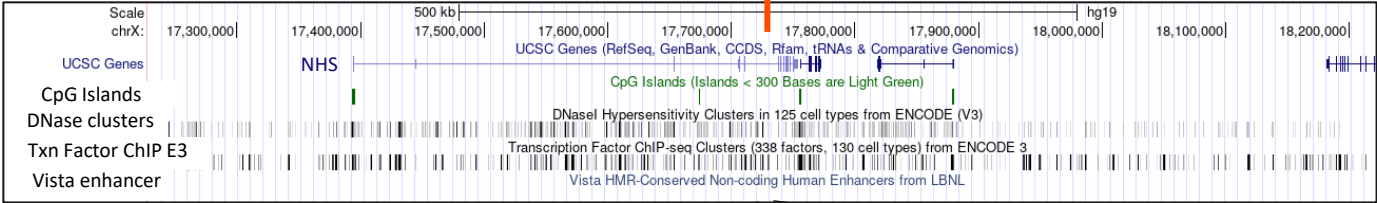

#2-A

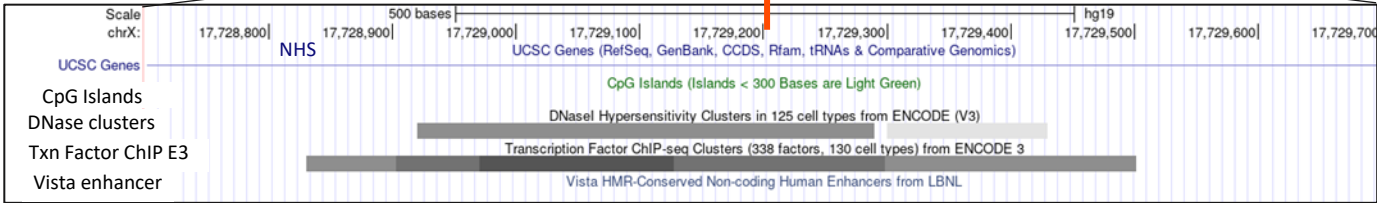

#3-C

#4-C

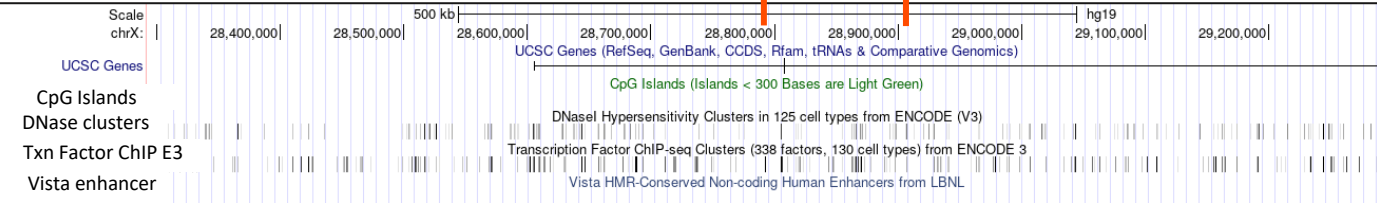

#3-C

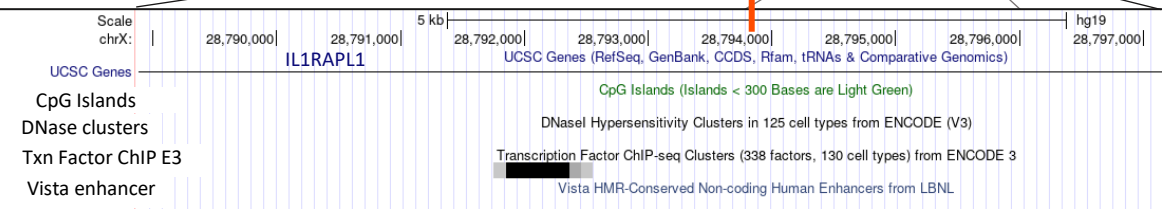

#4-C

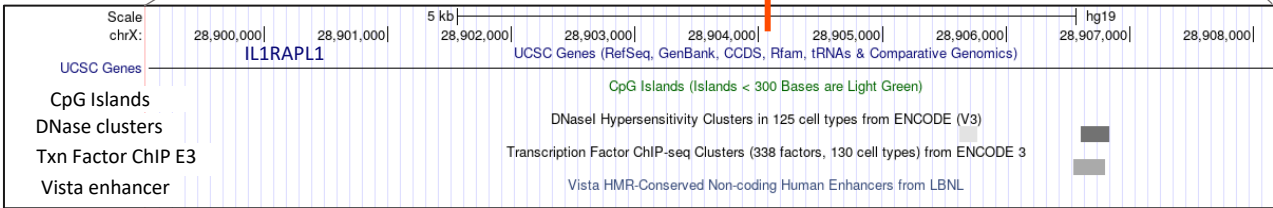

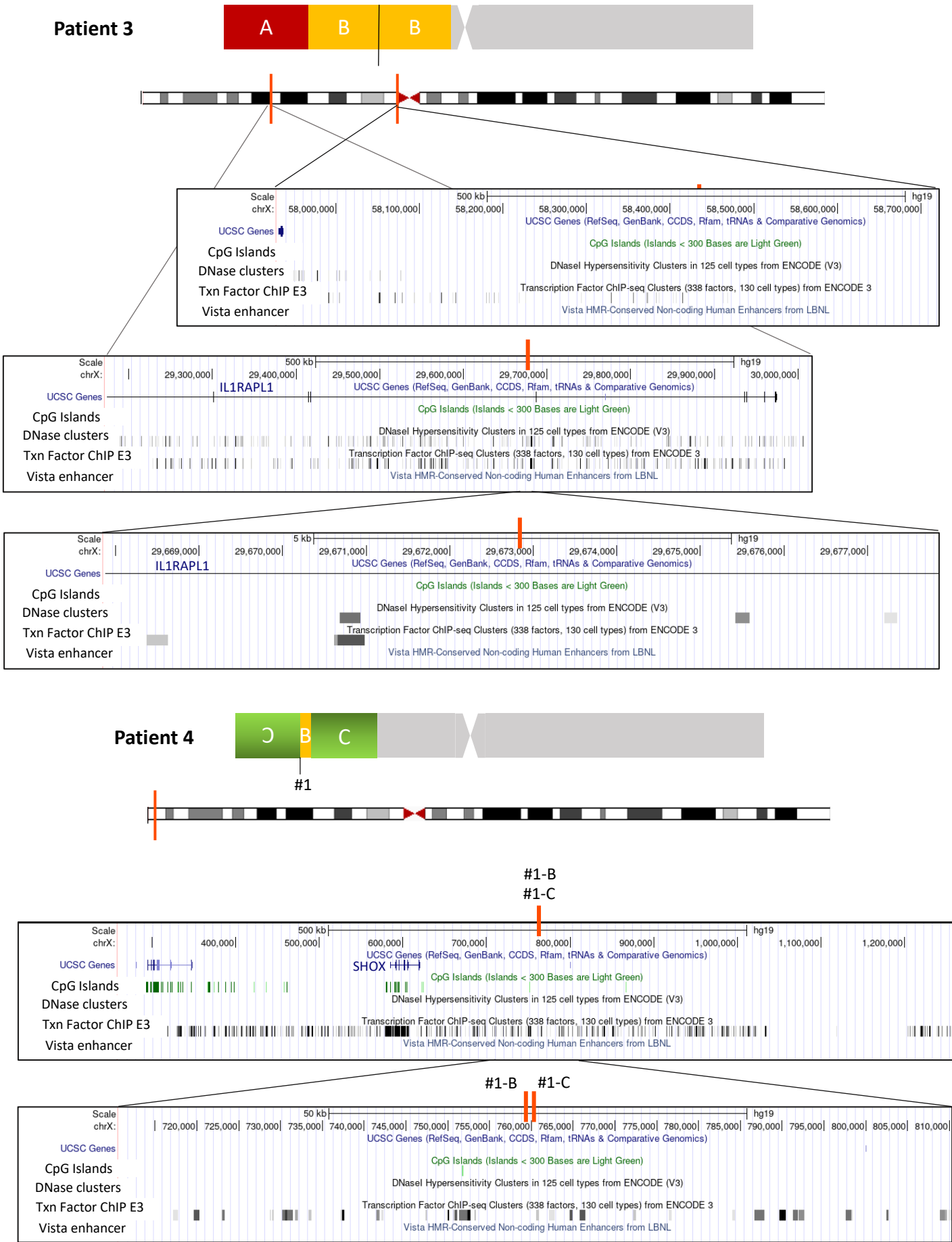

Supplement: Supplementary file 3 — Additional file 3: Figure S2. Breakpoint location. [file 13148_2021_1121_MOESM3_ESM.pdf]
