# Supplementary material for: Methylation status of genes escaping from X-chromosome inactivation in patients with X-chromosome rearrangements
Source: Clin Epigenetics. 2021 Jun 30;13:134. doi: 10.1186/s13148-021-01121-6 (PMC8244138; doi:10.1186/s13148-021-01121-6)
Supplement: Supplementary file 4 — Additional file 4: Figure S3. The number of escape genes. [file 13148_2021_1121_MOESM4_ESM.pdf]

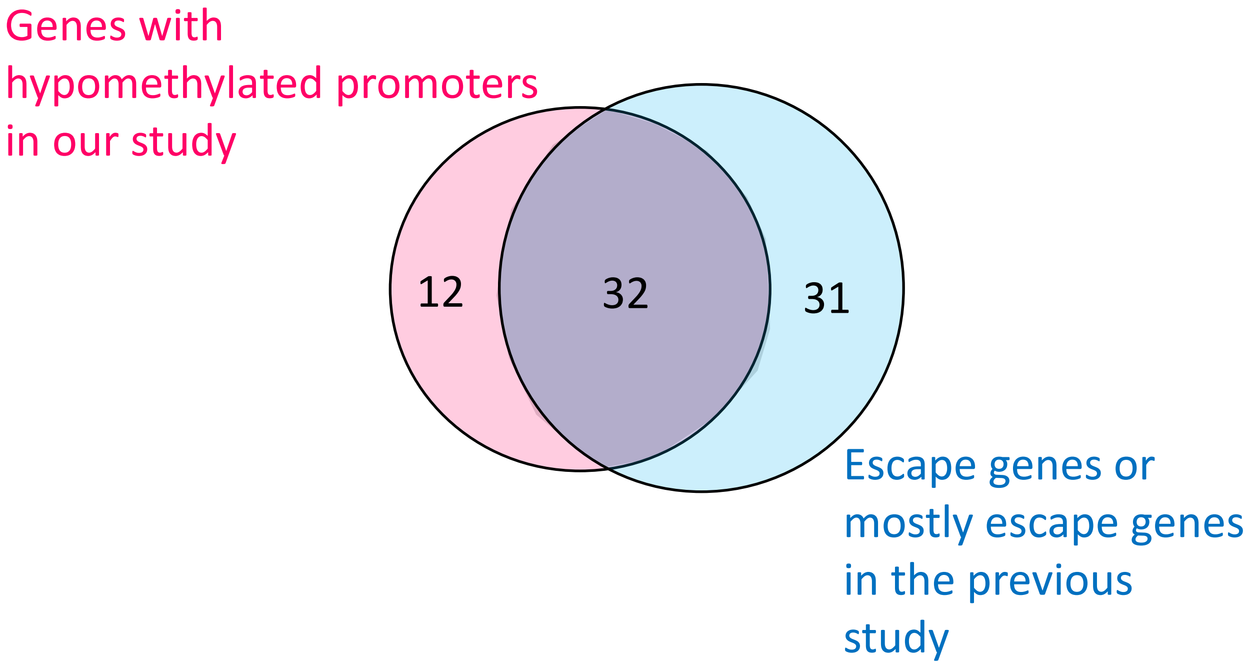

Figure S3. The number of escape genes. The pink circle indicates the number of genes with hypomethylated promoters in our study. The blue circle represents the number of escape genes or mostly escape genes in the previous study (1). The overlapped area shows the genes which we evaluated in the patients in this study.

Reference

1. Balaton BP, Cotton AM, Brown CJ. Derivation of consensus inactivation status for X-linked genes from genome-wide studies. Biol Sex Differ 2015;30:6:35.
