# Supplementary material for: Methylation status of genes escaping from X-chromosome inactivation in patients with X-chromosome rearrangements
Source: Clin Epigenetics. 2021 Jun 30;13:134. doi: 10.1186/s13148-021-01121-6 (PMC8244138; doi:10.1186/s13148-021-01121-6)
Supplement: Supplementary file 5 — Additional file 5: Table S2. The copy number of each escape gene in four patients. [file 13148_2021_1121_MOESM5_ESM.pdf]

Table S2. The copy number of each escape gene in four patients

Region	Analysis	Escape gene	Patient 1	Patient 2	Patient 3	Patient 4
PAR1	RRBS	*PLCXD1*	duplication	normal copy number	normal copy number	deletion
		PPP2R3B	duplication	normal copy number	normal copy number	deletion
		SHOX	duplication	normal copy number	normal copy number	deletion
		SLC25A6	duplication	normal copy number	normal copy number	duplication
		ASMTL	duplication	normal copy number	normal copy number	duplication
		AKAP17A	duplication	normal copy number	normal copy number	duplication
		CD99P1	duplication	normal copy number	normal copy number	duplication
		CD99	duplication	normal copy number	normal copy number	duplication
X-specific region	EPIC	*ARSD*	duplication	normal copy number	normal copy number	duplication
		MXRA5	duplication	normal copy number	normal copy number	duplication
		PRKX	duplication	normal copy number	normal copy number	duplication
		NLGN4X	duplication	normal copy number	normal copy number	duplication
		PNPLA4	duplication	normal copy number	normal copy number	duplication
		KAL1	duplication	normal copy number	normal copy number	duplication
		TCEANC	duplication	normal copy number	normal copy number	duplication
		RAB9A	duplication	normal copy number	normal copy number	duplication
		TRAPPC2	duplication	normal copy number	normal copy number	duplication
		GPM6B	duplication	normal copy number	normal copy number	duplication
		GEMIN8	duplication	normal copy number	normal copy number	duplication
		CA5BP1	duplication	normal copy number	normal copy number	duplication
		CA5B	duplication	normal copy number	normal copy number	duplication
		ZRAR2	duplication	normal copy number	normal copy number	duplication
		AP1S2	duplication	normal copy number	normal copy number	duplication
		CTPS2	duplication	normal copy number	normal copy number	duplication
		SYAP1	duplication	normal copy number	normal copy number	duplication
		TXLNG	duplication	normal copy number	normal copy number	duplication
		EIF1AX	normal copy number	duplication	normal copy number	duplication
		EIF2S3	normal copy number	duplication	normal copy number	duplication
		ZFX	normal copy number	duplication	normal copy number	duplication
		CXorf38	deletion	normal copy number	duplication	normal copy number
		DDX3X	deletion	normal copy number	duplication	normal copy number

FUNDC1	deletion	normal copy number	duplication	normal copy number
KDM6A	deletion	normal copy number	duplication	normal copy number
UBA1	normal copy number	normal copy number	duplication	normal copy number
CDK16	normal copy number	normal copy number	duplication	normal copy number
KDM5C	normal copy number	normal copy number	duplication	normal copy number
IQSEC2	normal copy number	normal copy number	duplication	normal copy number
RPS4X	normal copy number	normal copy number	normal copy number	normal copy number
JPX	normal copy number	normal copy number	normal copy number	normal copy number
L1CAM	deletion	normal copy number	normal copy number	normal copy number

PAR1, pseudoautosomal region 1; RRBS, Reduced Representation Bisulfite Sequencing

The three genes which showed abnormal methylation in Patient 1 are boldfaced.
