# Supplementary material for: Methylation status of genes escaping from X-chromosome inactivation in patients with X-chromosome rearrangements
Source: Clin Epigenetics. 2021 Jun 30;13:134. doi: 10.1186/s13148-021-01121-6 (PMC8244138; doi:10.1186/s13148-021-01121-6)
Supplement: Supplementary file 6 — Additional file 6: Table S3. The β value in promoter regions of escape genes in the patients. [file 13148_2021_1121_MOESM6_ESM.pdf]

Table S3. The β value in promoter regions of escape genes in the patients.

Genomic position	TargetID	Gene name	Female control			Patient 1	Patient 2	Patient 3	Patient 4
			Mean β value	Min βvalue	Max β value	βvalue	βvalue	βvalue	βvalue
2847478	cg05479376	ARSD	0.027	0.019	0.041	0.099	0.033	0.037	0.043
2847549	cg01337545	ARSD	0.137	0.086	0.203	0.263	0.145	0.134	0.107
2847557	cg21559133	ARSD	0.132	0.096	0.178	0.242	0.124	0.133	0.104
3264695	cg16141678	MXRA5	0.095	0.060	0.140	0.334	0.117	0.157	0.151
3264711	cg22698358	MXRA5	0.036	0.027	0.047	0.099	0.039	0.053	0.038
3264725	cg04371585	MXRA5	0.032	0.020	0.054	0.077	0.023	0.029	0.030
3264728	cg23832787	MXRA5	0.040	0.025	0.069	0.129	0.044	0.059	0.047
3264864	cg08414231	MXRA5	0.068	0.044	0.116	0.206	0.060	0.083	0.077
3631387	cg13879373	PRKX	0.030	0.021	0.038	0.100	0.046	0.057	0.051
3631575	cg04065558	PRKX	0.047	0.043	0.055	0.071	0.048	0.057	0.052
6145096	cg17811446	NLGN4X	0.091	0.059	0.121	0.159	0.086	0.128	0.115
6145346	cg20303283	NLGN4X	0.027	0.020	0.042	0.048	0.026	0.045	0.038
6145557	cg21875437	NLGN4X	0.015	0.005	0.027	0.017	0.011	0.024	0.012
6146608	cg24103438	NLGN4X	0.066	0.049	0.088	0.156	0.072	0.092	0.069
6146780	cg08881159	NLGN4X	0.046	0.035	0.058	0.067	0.043	0.067	0.051
6147113	cg15790913	NLGN4X	0.048	0.035	0.063	0.089	0.041	0.044	0.075
7895590	cg05508067	PNPLA4	0.041	0.021	0.086	0.222	0.067	0.061	0.056
7895607	cg01169463	PNPLA4	0.043	0.006	0.132	**0.395**	0.043	0.065	0.055
7895754	cg01823238	PNPLA4	0.050	0.019	0.136	**0.286**	0.063	0.061	0.063
7895900	cg21864248	PNPLA4	0.118	0.099	0.168	0.183	0.116	0.175	0.109
8699351	cg05152051	KAL1	0.053	0.034	0.065	0.136	0.081	0.078	0.076
8699462	cg08059058	KAL1	0.071	0.044	0.123	0.163	0.055	0.071	0.063
8700315	cg04084455	KAL1	0.033	0.019	0.063	0.105	0.027	0.048	0.036
8700341	cg02156712	KAL1	0.024	0.013	0.033	0.066	0.025	0.032	0.025
8700400	cg10088365	KAL1	0.038	0.019	0.062	0.191	0.047	0.055	0.052
8700496	cg25389127	KAL1	0.048	0.036	0.073	0.111	0.054	0.047	0.051
13671130	cg17279959	TCEANC	0.087	0.029	0.210	**0.394**	0.072	0.088	0.128
13671259	cg24882621	TCEANC	0.070	0.031	0.153	**0.405**	0.091	0.086	0.159
13671272	cg16251333	TCEANC	0.072	0.029	0.183	**0.344**	0.069	0.070	0.073
13707062	cg14621531	RAB9A	0.063	0.040	0.081	0.264	0.083	0.097	0.090
13707135	cg02723311	RAB9A	0.024	0.014	0.049	0.063	0.021	0.034	0.024
13707694	cg02620228	RAB9A	0.100	0.051	0.149	0.260	0.201	0.176	0.173
13752813	cg17512860	TRAPPC2	0.081	0.051	0.172	0.331	0.081	0.128	0.086
13752958	cg23353205	TRAPPC2	0.042	0.029	0.068	0.175	0.064	0.079	0.045
13753119	cg05598468	TRAPPC2	0.081	0.051	0.191	0.186	0.037	0.089	0.040
13753381	cg14962776	TRAPPC2	0.049	0.029	0.067	0.185	0.053	0.084	0.085

13956720	cg01310267	GPM6B	0.066	0.028	0.155	**0.352**	0.063	0.094	0.094
13957159	cg18776021	GPM6B	0.121	0.076	0.218	**0.335**	0.141	0.098	0.210
14047762	cg19233518	GEMIN8	0.092	0.064	0.175	0.219	0.087	0.061	0.079
14048019	cg13675330	GEMIN8	0.031	0.016	0.076	0.189	0.036	0.031	0.028
14048191	cg21239691	GEMIN8	0.041	0.022	0.096	0.243	0.046	0.057	0.040
14048197	cg07413830	GEMIN8	0.050	0.025	0.118	0.270	0.049	0.047	0.060
14547320	cg24596823	GLRA2	0.078	0.055	0.108	0.090	0.064	0.068	0.068
14547594	cg03037266	GLRA2	0.122	0.083	0.151	0.305	0.166	0.198	0.198
15692898	cg08211022	CA5BP	0.096	0.071	0.123	0.144	0.095	0.093	0.079
15692909	cg15926952	CA5BP	0.086	0.057	0.107	0.159	0.059	0.108	0.066
15693037	cg10444261	CA5BP	0.036	0.024	0.049	0.123	0.043	0.030	0.032
15693238	cg26422761	CA5BP	0.035	0.024	0.049	0.127	0.034	0.034	0.040
15693836	cg26816294	CA5BP	0.055	0.033	0.083	0.041	0.067	0.030	0.041
15693956	cg22139325	CA5BP	0.039	0.026	0.050	0.120	0.040	0.044	0.044
15756062	cg22702707	CA5B	0.097	0.072	0.117	0.152	0.082	0.116	0.093
15756384	cg18430208	CA5B	0.036	0.029	0.044	0.133	0.086	0.105	0.073
15756407	cg25954223	CA5B	0.061	0.049	0.076	0.111	0.048	0.067	0.063
15808073	cg22042328	ZRSR2	0.052	0.031	0.076	0.043	0.036	0.040	0.043
15808320	cg13274727	ZRSR2	0.047	0.034	0.060	0.055	0.042	0.049	0.053
15808408	cg13552866	ZRSR2	0.043	0.034	0.054	0.050	0.056	0.045	0.045
15808692	cg19535369	ZRSR2	0.072	0.052	0.090	0.066	0.051	0.088	0.055
15872951	cg17659886	AP1S2	0.033	0.025	0.044	0.060	0.039	0.038	0.057
15873314	cg08048775	AP1S2	0.032	0.025	0.042	0.031	0.026	0.040	0.034
15873337	cg05106463	AP1S2	0.028	0.017	0.033	0.032	0.022	0.035	0.020
15873351	cg05419061	AP1S2	0.019	0.014	0.025	0.034	0.028	0.016	0.026
15873481	cg25100404	AP1S2	0.027	0.017	0.034	0.048	0.038	0.033	0.035
16730380	cg05572834	CTPS2	0.103	0.068	0.138	0.148	0.119	0.118	0.132
16730680	cg07079445	CTPS2	0.061	0.047	0.070	0.079	0.066	0.057	0.069
16730881	cg01732428	CTPS2	0.077	0.058	0.101	0.100	0.065	0.107	0.093
16730923	cg19388636	CTPS2	0.062	0.043	0.086	0.080	0.040	0.062	0.044
16737422	cg22249566	SYAP1	0.096	0.069	0.120	0.149	0.107	0.151	0.084
16737521	cg05908833	SYAP1	0.047	0.034	0.064	0.049	0.036	0.024	0.041
16737621	cg27351642	SYAP1	0.057	0.046	0.075	0.062	0.068	0.039	0.055
16737686	cg25793649	SYAP1	0.028	0.020	0.040	0.025	0.030	0.035	0.032
16737692	cg08818886	SYAP1	0.013	0.006	0.018	0.013	0.014	0.016	0.011
16737800	cg00331433	SYAP1	0.036	0.028	0.047	0.045	0.038	0.030	0.036
16804362	cg27636290	CXorf15	0.031	0.024	0.040	0.033	0.022	0.041	0.020
16804394	cg11720142	CXorf15	0.038	0.025	0.055	0.055	0.050	0.056	0.044
16804461	cg03348338	CXorf15	0.106	0.090	0.126	0.105	0.117	0.141	0.097

16804636	cg22917082	CXorf15	0.037	0.021	0.048	0.048	0.039	0.037	0.045
20160069	cg14354749	EIF1AX	0.036	0.009	0.060	0.038	0.033	0.023	0.030
20160086	cg22264584	EIF1AX	0.040	0.003	0.066	0.036	0.014	0.024	0.020
24072448	cg00914804	EIF2S3	0.050	0.031	0.120	0.093	0.039	0.050	0.050
24072640	cg09835024	EIF2S3	0.058	0.026	0.116	0.050	0.038	0.059	0.044
24167840	cg07639739	ZFX	0.074	0.057	0.092	0.081	0.089	0.094	0.062
24168810	cg03159836	ZFX	0.030	0.015	0.041	0.023	0.039	0.035	0.021
40506477	cg24085719	CXorf38	0.055	0.026	0.131	0.036	0.067	0.029	0.106
40506804	cg15322570	CXorf38	0.066	0.020	0.162	0.057	0.062	0.049	0.260
40506974	cg22148243	CXorf38	0.035	0.018	0.084	0.029	0.020	0.035	0.040
40506981	cg03169432	CXorf38	0.023	0.017	0.031	0.029	0.035	0.027	0.037
41192164	cg12689375	DDX3X	0.032	0.022	0.055	0.027	0.037	0.036	0.044
41192872	cg27042767	DDX3X	0.035	0.026	0.047	0.033	0.039	0.042	0.034
41193241	cg11251991	DDX3X	0.030	0.023	0.039	0.056	0.060	0.039	0.056
41193326	cg16117744	DDX3X	0.034	0.026	0.050	0.044	0.036	0.025	0.043
44401762	cg14084176	FUNDC1	0.057	0.039	0.081	0.070	0.077	0.064	0.120
44402314	cg21465470	FUNDC1	0.079	0.058	0.113	0.091	0.072	0.083	0.069
44402332	cg01573544	FUNDC1	0.026	0.017	0.041	0.047	0.022	0.030	0.022
44402383	cg04565250	FUNDC1	0.048	0.018	0.119	0.089	0.045	0.052	0.051
44402448	cg18511445	FUNDC1	0.094	0.062	0.162	0.106	0.117	0.124	0.121
44731813	cg15923127	KDM6A	0.032	0.025	0.045	0.032	0.023	0.044	0.027
44732352	cg07167981	KDM6A	0.024	0.016	0.033	0.035	0.018	0.025	0.028
44732417	cg06877198	KDM6A	0.047	0.039	0.059	0.049	0.043	0.042	0.049
44732741	cg24355643	KDM6A	0.017	0.006	0.026	0.019	0.025	0.010	0.021
44732817	cg00751785	KDM6A	0.023	0.004	0.029	0.047	0.021	0.018	0.030
44733206	cg03267667	KDM6A	0.025	0.009	0.036	0.026	0.021	0.026	0.009
47050029	cg25059428	UBA1	0.122	0.082	0.175	0.119	0.167	0.125	0.098
47050296	cg22470298	UBA1	0.100	0.060	0.138	0.098	0.055	0.085	0.157
47053031	cg03149049	UBA1	0.014	0.006	0.021	0.013	0.010	0.020	0.013
47053154	cg08341987	UBA1	0.035	0.024	0.047	0.064	0.075	0.088	0.056
47053156	cg22268449	UBA1	0.037	0.022	0.047	0.043	0.055	0.051	0.072
47053161	cg17699837	UBA1	0.027	0.021	0.035	0.022	0.020	0.034	0.023
47053220	cg06286431	UBA1	0.027	0.017	0.039	0.018	0.020	0.027	0.027
47077487	cg13907504	CDK16	0.142	0.057	0.233	0.344	0.481	0.216	0.464
47077877	cg11333230	CDK16	0.095	0.060	0.153	0.116	0.115	0.067	0.099
47077930	cg23212388	CDK16	0.097	0.046	0.141	0.147	0.200	0.084	0.216
47077986	cg22826577	CDK16	0.141	0.088	0.183	0.219	0.227	0.134	0.219
53254132	cg09554224	KDM5C	0.037	0.028	0.048	0.036	0.036	0.050	0.035
53254503	cg01363850	KDM5C	0.019	0.013	0.027	0.019	0.024	0.028	0.016

53254653	cg04927982	KDM5C	0.023	0.015	0.029	0.015	0.012	0.025	0.019
53254760	cg12234996	KDM5C	0.050	0.025	0.069	0.073	0.043	0.054	0.071
53350672	cg11670235	IQSEC2	0.068	0.048	0.089	0.087	0.062	0.056	0.073
53350693	cg25849068	IQSEC2	0.043	0.021	0.082	0.100	0.044	0.063	0.074
53350895	cg13367883	IQSEC2	0.128	0.080	0.196	0.278	0.152	0.239	0.357
71496641	cg06868150	RPS4X	0.057	0.035	0.076	0.032	0.035	0.036	0.029
71496665	cg25869832	RPS4X	0.023	0.005	0.041	0.035	0.007	0.027	0.059
71496701	cg01714671	RPS4X	0.057	0.046	0.067	0.057	0.062	0.070	0.107
71497035	cg08859156	RPS4X	0.045	0.038	0.057	0.032	0.040	0.043	0.066
71497208	cg03273228	RPS4X	0.121	0.112	0.136	0.147	0.133	0.138	0.185
71497224	cg02546818	RPS4X	0.022	0.013	0.030	0.023	0.022	0.047	0.094
71497226	cg27111711	RPS4X	0.077	0.066	0.092	0.079	0.078	0.064	0.048
73164102	cg17405188	LOC554203	0.035	0.025	0.048	0.016	0.032	0.032	0.037
73164228	cg01752030	LOC554203	0.040	0.030	0.052	0.048	0.038	0.034	0.044
73164390	cg09803537	LOC554203	0.054	0.040	0.071	0.053	0.043	0.072	0.065
153140985	cg18812946	L1CAM	0.119	0.076	0.168	0.082	0.062	0.104	0.087
153141270	cg06300944	L1CAM	0.093	0.053	0.135	0.041	0.065	0.114	0.100

A yellow background means probes in duplicated regions. A pale blue background means probes in deleted regions. When the βvalue is higher than 0.25, the βvalue is shown in red letters. When more than two consecutive probes' βvalue within 1,000 bp from TSS of a gene is higher than 0.25, the βvalue is shown in red bold letters.
