# Supplementary material for: Methylation status of genes escaping from X-chromosome inactivation in patients with X-chromosome rearrangements
Source: Clin Epigenetics. 2021 Jun 30;13:134. doi: 10.1186/s13148-021-01121-6 (PMC8244138; doi:10.1186/s13148-021-01121-6)
Supplement: Supplementary file 7 — Additional file 7: Table S4. The β value in promoter regions of inactivated genes in the patients. [file 13148_2021_1121_MOESM7_ESM.pdf]

Table S4. The β value in promoter regions of inactivated genes in the patients.

Genomic position	TargetID	Gene name	Male control		Female				Patient 1	Patient 2	Patient 3	Patient 4
			Mean βvalue	Max β value	Mean βvalue	Min β value	Max β value	Max β value+0.05	βvalue	βvalue	βvalue	βvalue
9983823	cg00689116	WWC3	0.020	0.026	0.406	0.332	0.480	0.530	0.549	0.497	0.349	0.369
9984045	cg25270201	WWC3	0.077	0.138	0.409	0.311	0.512	0.562	0.547	0.450	0.390	0.408
11445762	cg02969243	ARHGAP6	0.083	0.107	0.337	0.285	0.445	0.495	0.476	0.407	0.440	0.378
11446093	cg08587977	ARHGAP6	0.021	0.031	0.354	0.301	0.419	0.469	**0.539**	0.367	0.375	0.474
11446095	cg25873934	ARHGAP6	0.026	0.031	0.419	0.371	0.494	0.544	**0.549**	0.466	0.422	0.504
11683131	cg13868987	ARHGAP6	0.102	0.146	0.382	0.341	0.436	0.486	0.442	0.437	0.393	0.402
11683625	cg18036967	ARHGAP6	0.032	0.048	0.367	0.305	0.440	0.490	0.468	0.400	0.322	0.346
11683838	cg23783361	ARHGAP6	0.080	0.100	0.413	0.374	0.463	0.513	0.497	0.404	0.386	0.438
11683977	cg07828380	ARHGAP6	0.128	0.147	0.460	0.416	0.504	0.554	0.524	0.498	0.488	0.474
12809100	cg27325673	PRPS2	0.026	0.050	0.414	0.335	0.522	0.572	0.599	0.499	0.433	0.511
12809173	cg26190455	PRPS2	0.016	0.024	0.384	0.330	0.455	0.505	0.471	0.388	0.339	0.421
12809328	cg01669374	PRPS2	0.062	0.106	0.392	0.281	0.531	0.581	0.552	0.351	0.449	0.474
12809589	cg18492262	PRPS2	0.044	0.055	0.342	0.264	0.447	0.497	0.479	0.311	0.305	0.377
15353857	cg12194828	PIGA	0.048	0.059	0.405	0.343	0.471	0.521	0.467	0.320	0.376	0.382
15353925	cg05275343	PIGA	0.031	0.043	0.376	0.326	0.434	0.484	0.549	0.393	0.411	0.476
18372126	cg09799350	SCML2	0.027	0.033	0.422	0.326	0.497	0.547	0.458	0.408	0.360	0.485
18372963	cg08300622	SCML2	0.034	0.046	0.464	0.399	0.537	0.587	0.474	0.470	0.411	0.529
21392324	cg05444994	CNKSR2	0.034	0.045	0.458	0.369	0.531	0.581	0.537	0.451	0.475	0.452
21392334	cg13616735	CNKSR2	0.026	0.033	0.393	0.333	0.442	0.492	0.486	0.393	0.403	0.431
21392642	cg19807373	CNKSR2	0.019	0.025	0.416	0.350	0.476	0.526	0.492	0.430	0.408	0.445
21392644	cg09252204	CNKSR2	0.022	0.031	0.382	0.283	0.465	0.515	0.433	0.379	0.334	0.374
21392988	cg26267038	CNKSR2	0.057	0.072	0.455	0.377	0.511	0.561	0.514	0.459	0.526	0.517
21393306	cg05747459	CNKSR2	0.105	0.140	0.439	0.382	0.519	0.569	0.499	0.473	0.366	0.523
23761589	cg26207017	ACOT9	0.027	0.033	0.401	0.343	0.480	0.530	0.380	0.368	0.347	0.417
23761651	cg24123780	ACOT9	0.074	0.111	0.442	0.368	0.499	0.549	0.433	0.486	0.429	0.412
23761736	cg08289016	ACOT9	0.133	0.174	0.458	0.395	0.525	0.575	0.413	0.465	0.405	0.413
23801440	cg09725439	SAT1	0.021	0.027	0.430	0.380	0.475	0.525	0.420	0.386	0.479	0.403
23801656	cg14550383	SAT1	0.060	0.081	0.473	0.392	0.525	0.575	0.582	0.476	0.501	0.532
24665506	cg15259889	PCYT1B	0.022	0.034	0.346	0.273	0.380	0.430	0.474	0.437	0.422	0.456
24665508	cg17627526	PCYT1B	0.023	0.032	0.344	0.309	0.381	0.431	0.439	0.403	0.367	0.388
24665655	cg21337832	PCYT1B	0.047	0.065	0.419	0.357	0.480	0.530	0.463	0.535	0.482	0.455
30326676	cg00040455	NR0B1	0.078	0.118	0.387	0.330	0.472	0.522	0.447	0.378	0.466	0.345
30327110	cg11869614	NR0B1	0.053	0.070	0.499	0.453	0.565	0.615	0.574	0.540	0.632	0.591
30327487	cg23208903	NR0B1	0.071	0.086	0.444	0.388	0.517	0.567	0.450	0.422	0.415	0.455
30327545	cg07820420	NR0B1	0.083	0.117	0.324	0.289	0.388	0.438	0.406	0.264	0.351	0.372
30327547	cg13012525	NR0B1	0.091	0.113	0.381	0.324	0.448	0.498	0.425	0.381	0.358	0.450
30327615	cg10291990	NR0B1	0.047	0.059	0.429	0.367	0.469	0.519	0.458	0.394	0.469	0.436
30906902	cg09319393	MAP3K7IP3	0.052	0.069	0.397	0.358	0.439	0.489	0.427	0.397	0.400	0.350
30907117	cg20372745	MAP3K7IP3	0.042	0.052	0.455	0.362	0.509	0.559	0.543	0.436	0.485	0.445
30907598	cg25416923	MAP3K7IP3	0.070	0.102	0.338	0.298	0.371	0.421	0.433	0.284	0.372	0.333
37430711	cg02166322	LANCL3	0.029	0.043	0.370	0.295	0.416	0.466	0.391	0.368	0.363	0.413
37430889	cg21882403	LANCL3	0.049	0.063	0.459	0.414	0.493	0.543	0.512	0.393	0.427	0.439
41781902	cg09815801	CASK	0.024	0.033	0.421	0.319	0.470	0.520	**0.076**	0.362	0.380	0.370
41782111	cg14622782	CASK	0.069	0.114	0.345	0.270	0.436	0.486	**0.079**	0.247	0.250	0.232
41782322	cg17548613	CASK	0.101	0.135	0.465	0.360	0.537	0.587	**0.136**	0.497	0.436	0.401

41782695	cg03983969	CASK	0.024	0.035	0.473	0.420	0.534	0.584	**0.086**	0.412	0.436	0.430
47004051	cg02165720	NDUFB11	0.026	0.046	0.491	0.444	0.560	0.610	0.500	0.496	0.487	0.604
47004107	cg05418506	NDUFB11	0.016	0.024	0.458	0.394	0.516	0.566	0.467	0.356	0.459	0.448
47004146	cg02538330	NDUFB11	0.021	0.029	0.426	0.327	0.495	0.545	0.433	0.423	0.442	0.373
47341898	cg02124059	ZNF41	0.120	0.150	0.499	0.442	0.540	0.590	0.490	0.474	0.596	0.435
47342108	cg09020104	ZNF41	0.016	0.022	0.313	0.263	0.368	0.418	0.358	0.301	0.335	0.337
47342124	cg05526804	ZNF41	0.023	0.034	0.357	0.318	0.435	0.485	0.391	0.349	0.479	0.363
47441581	cg02896361	SYN1	0.080	0.107	0.447	0.389	0.506	0.556	0.439	0.402	0.401	0.434
47441605	cg18009325	SYN1	0.092	0.114	0.384	0.314	0.463	0.513	0.430	0.325	0.418	0.361
47695826	cg19580826	ZNF81	0.075	0.098	0.439	0.394	0.474	0.524	0.472	0.384	0.487	0.399
47695967	cg11773570	ZNF81	0.027	0.042	0.404	0.358	0.454	0.504	0.357	0.334	0.496	0.377
48367193	cg08785133	PORCN	0.044	0.050	0.468	0.412	0.514	0.564	0.551	0.471	0.557	0.443
48367513	cg03244432	PORCN	0.018	0.025	0.372	0.308	0.410	0.460	0.382	0.373	0.384	0.292
48755154	cg27403913	PQBP1	0.020	0.024	0.401	0.338	0.481	0.531	0.475	0.405	0.417	0.444
48755433	cg21364772	PQBP1	0.034	0.046	0.383	0.351	0.417	0.467	0.316	0.306	0.379	0.269
48769307	cg01488378	SLC35A2	0.041	0.054	0.426	0.351	0.532	0.582	0.531	0.394	0.448	0.427
48769370	cg14132995	SLC35A2	0.044	0.067	0.376	0.302	0.483	0.533	0.374	0.337	0.393	0.325
48776349	cg03692228	PIM2	0.029	0.039	0.379	0.298	0.457	0.507	0.457	0.431	0.369	0.367
48776465	cg17192356	PIM2	0.061	0.090	0.400	0.307	0.471	0.521	0.488	0.352	0.482	0.365
48776567	cg12888005	PIM2	0.055	0.103	0.492	0.450	0.560	0.610	0.473	0.436	0.616	0.529
48776812	cg21267792	PIM2	0.034	0.046	0.379	0.285	0.449	0.499	0.439	0.397	0.469	0.490
48815581	cg03835117	OTUD5	0.030	0.040	0.343	0.286	0.390	0.440	0.387	0.325	0.412	0.436
48815643	cg12591117	OTUD5	0.056	0.111	0.395	0.316	0.459	0.509	0.613	0.452	0.470	0.446
48815856	cg02480419	OTUD5	0.037	0.055	0.481	0.413	0.531	0.581	0.497	0.470	0.555	0.448
48828399	cg03341064	KCND1	0.083	0.131	0.437	0.377	0.486	0.536	0.512	0.423	0.384	0.409
48828779	cg25676950	KCND1	0.028	0.038	0.379	0.320	0.434	0.484	0.367	0.382	0.341	0.479
48858660	cg16930349	GRIPAP1	0.103	0.135	0.408	0.329	0.479	0.529	0.424	0.348	0.410	0.383
48858679	cg04703500	GRIPAP1	0.044	0.061	0.461	0.392	0.511	0.561	0.466	0.385	0.491	0.438
48858686	cg13443410	GRIPAP1	0.040	0.066	0.481	0.397	0.583	0.633	0.473	0.423	0.506	0.389
48858832	cg15392343	GRIPAP1	0.043	0.053	0.462	0.396	0.499	0.549	0.476	0.367	0.478	0.381
48931388	cg20371650	PRAF2	0.028	0.037	0.443	0.391	0.505	0.555	0.451	0.467	0.572	0.464
48931623	cg11286196	PRAF2	0.031	0.040	0.413	0.377	0.446	0.496	0.436	0.379	0.479	0.411
48980043	cg01150083	GPKOW	0.046	0.057	0.454	0.364	0.512	0.562	0.436	0.422	0.424	0.311
48980092	cg07240096	GPKOW	0.028	0.038	0.425	0.360	0.492	0.542	0.481	0.407	0.499	0.482
49028673	cg17878135	PLP2	0.030	0.043	0.420	0.346	0.548	0.598	0.490	0.476	0.508	0.488
49029104	cg07210835	PLP2	0.093	0.126	0.435	0.367	0.525	0.575	0.453	0.341	0.508	0.427
49687306	cg00264378	CLCN5	0.025	0.037	0.468	0.427	0.524	0.574	0.545	0.435	0.512	0.483
49687331	cg21911363	CLCN5	0.033	0.048	0.428	0.385	0.468	0.518	0.470	0.425	0.515	0.439
50557114	cg22750156	SHROOM4	0.035	0.045	0.471	0.426	0.514	0.564	0.507	0.485	0.434	0.492
50557436	cg05145435	SHROOM4	0.088	0.120	0.415	0.358	0.497	0.547	0.408	0.460	0.422	0.508
51075046	cg00648125	NUDT10	0.064	0.088	0.447	0.400	0.513	0.563	0.645	0.666	0.557	0.658
51075126	cg02975846	NUDT10	0.075	0.103	0.344	0.292	0.453	0.503	0.320	0.254	0.323	0.409
51075273	cg20430749	NUDT10	0.064	0.087	0.384	0.337	0.427	0.477	0.418	0.366	0.447	0.397
51546668	cg01195014	MAGED1	0.050	0.082	0.449	0.352	0.497	0.547	0.464	0.382	0.373	0.403
51636568	cg00597858	MAGED1	0.027	0.038	0.345	0.307	0.397	0.447	0.393	0.257	0.318	0.341
51636629	cg03742148	MAGED1	0.034	0.057	0.366	0.276	0.420	0.470	0.396	0.342	0.279	0.376
53461213	cg04241572	HSD17B10	0.028	0.040	0.328	0.272	0.416	0.466	0.402	0.299	0.296	0.435
53461386	cg26323797	HSD17B10	0.128	0.158	0.485	0.381	0.586	0.636	0.624	0.406	0.463	0.540

54069630	cg23245763	PHF8	0.041	0.068	0.420	0.298	0.526	0.576	0.624	0.467	0.555	0.403
54069785	cg01961587	PHF8	0.040	0.052	0.430	0.379	0.501	0.551	0.376	0.396	0.499	0.532
54069813	cg18626978	PHF8	0.018	0.027	0.384	0.311	0.468	0.518	0.378	0.344	0.365	0.490
54209709	cg04326875	FAM120C	0.041	0.057	0.417	0.368	0.500	0.550	0.433	0.393	0.519	0.369
54209920	cg13183496	FAM120C	0.025	0.032	0.471	0.411	0.518	0.568	0.516	0.423	0.560	0.474
54384161	cg15048430	WNK3	0.053	0.061	0.442	0.378	0.500	0.550	0.486	0.422	0.423	0.442
54384719	cg16235803	WNK3	0.038	0.053	0.403	0.311	0.497	0.547	0.464	0.389	0.552	0.502
54384964	cg12707233	WNK3	0.044	0.059	0.459	0.413	0.508	0.558	0.431	0.462	0.556	0.538
54385394	cg02896496	WNK3	0.019	0.028	0.455	0.420	0.502	0.552	0.535	0.502	0.513	0.468
54834292	cg07767690	MAGED2	0.016	0.021	0.414	0.380	0.473	0.523	0.350	0.431	0.458	0.410
54835315	cg22674664	MAGED2	0.041	0.046	0.487	0.426	0.552	0.602	0.505	0.483	0.599	0.476
54835382	cg22660483	MAGED2	0.039	0.059	0.478	0.418	0.537	0.587	0.479	0.404	0.557	0.574
54947373	cg06942445	TRO	0.027	0.038	0.396	0.352	0.445	0.495	0.385	0.345	0.422	0.371
54947822	cg23360535	TRO	0.048	0.066	0.436	0.391	0.474	0.524	0.413	0.384	0.443	0.484
55026485	cg05961595	APEX2	0.024	0.032	0.360	0.297	0.413	0.463	0.423	0.323	0.386	0.391
55026807	cg26927606	APEX2	0.041	0.050	0.483	0.441	0.521	0.571	0.498	0.470	0.550	0.485
55026811	cg13311758	APEX2	0.036	0.049	0.461	0.422	0.505	0.555	0.442	0.434	0.518	0.406
55478180	cg18869368	MAGEH1	0.040	0.057	0.338	0.271	0.428	0.478	0.317	0.327	0.393	0.261
55478329	cg04029630	MAGEH1	0.032	0.048	0.471	0.386	0.508	0.558	0.599	0.521	0.643	0.585
55515653	cg00834229	USP51	0.032	0.046	0.399	0.350	0.487	0.537	0.493	0.347	0.537	0.502
55515720	cg01084827	USP51	0.060	0.081	0.356	0.290	0.417	0.467	0.449	0.400	0.481	0.462
56258808	cg22829182	KLF8	0.033	0.050	0.371	0.319	0.427	0.477	0.404	0.390	0.450	0.570
56259040	cg03610137	KLF8	0.036	0.048	0.439	0.397	0.481	0.531	0.483	0.423	0.527	0.430
57937045	cg25930738	ZXDA	0.036	0.048	0.371	0.327	0.415	0.465	0.333	0.269	0.390	0.250
57937143	cg09249608	ZXDA	0.106	0.166	0.444	0.371	0.578	0.628	0.296	0.293	0.489	0.307
62570990	cg09070199	SPIN4	0.020	0.026	0.436	0.320	0.507	0.557	0.441	0.417	0.515	0.331
62571359	cg09022442	SPIN4	0.015	0.022	0.419	0.360	0.500	0.550	0.489	0.424	0.468	0.373
62780753	cg18625305	LOC92249	0.031	0.040	0.356	0.274	0.428	0.478	0.344	0.330	0.489	0.313
62780793	cg16956903	LOC92249	0.062	0.087	0.349	0.281	0.447	0.497	0.353	0.252	0.341	0.249
62780844	cg05375065	LOC92249	0.050	0.064	0.485	0.416	0.595	0.645	0.473	0.418	0.430	0.365
62780853	cg14314451	LOC92249	0.029	0.038	0.475	0.438	0.523	0.573	0.481	0.448	0.544	0.417
62975065	cg00727483	ARHGEF9	0.021	0.029	0.443	0.365	0.523	0.573	0.473	0.454	0.551	0.343
62975151	cg15917786	ARHGEF9	0.027	0.038	0.425	0.363	0.479	0.529	0.392	0.370	0.413	0.452
62975162	cg13489005	ARHGEF9	0.020	0.024	0.439	0.371	0.499	0.549	0.469	0.389	0.461	0.491
64196367	cg06327799	ZC4H2	0.042	0.058	0.331	0.276	0.434	0.484	0.325	0.301	0.315	0.379
64196429	cg16359563	ZC4H2	0.037	0.045	0.322	0.283	0.376	0.426	0.289	0.383	0.287	0.365
64196642	cg27326620	ZC4H2	0.101	0.137	0.391	0.299	0.501	0.551	0.437	0.357	0.516	0.434
64196751	cg11049305	ZC4H2	0.052	0.061	0.361	0.277	0.444	0.494	0.336	0.367	0.341	0.328
64754624	cg17299883	LAS1L	0.061	0.094	0.435	0.340	0.562	0.612	0.433	0.289	0.384	0.289
64754863	cg17001703	LAS1L	0.037	0.043	0.498	0.458	0.543	0.593	0.517	0.480	0.581	0.557
66763696	cg19102572	AR	0.070	0.088	0.392	0.352	0.433	0.483	0.368	0.338	0.363	0.331
66764000	cg16679837	AR	0.083	0.114	0.481	0.406	0.556	0.606	0.510	0.468	0.411	0.353
67718493	cg12039967	YIPF6	0.035	0.050	0.465	0.426	0.515	0.565	0.526	0.534	0.587	0.495
67718700	cg03939693	YIPF6	0.027	0.040	0.343	0.270	0.415	0.465	0.350	0.414	0.300	0.310
67718716	cg17203703	YIPF6	0.051	0.079	0.470	0.385	0.598	0.648	0.582	0.422	0.436	0.372
67718729	cg19605909	YIPF6	0.079	0.105	0.469	0.433	0.538	0.588	0.529	0.428	0.459	0.442
67913300	cg08728127	STARD8	0.113	0.155	0.331	0.294	0.387	0.437	0.391	0.269	0.352	0.264
67913327	cg24149051	STARD8	0.104	0.142	0.392	0.328	0.450	0.500	0.450	0.435	0.466	0.361

68836202	cg12137402	EDA	0.026	0.033	0.476	0.421	0.528	0.578	0.517	0.505	0.489	0.437
68836571	cg06126323	EDA	0.072	0.093	0.414	0.345	0.500	0.550	0.492	0.404	0.526	0.479
69664482	cg20051589	DLG3	0.072	0.101	0.311	0.269	0.378	0.428	0.325	0.309	0.189	0.252
69664580	cg24658899	DLG3	0.059	0.072	0.418	0.349	0.478	0.528	0.464	0.362	0.288	0.410
69664599	cg03894002	DLG3	0.076	0.099	0.467	0.438	0.523	0.573	0.502	0.423	0.447	0.478
69664757	cg16394734	DLG3	0.130	0.145	0.485	0.459	0.523	0.573	0.504	0.488	0.440	0.436
69665118	cg23429746	DLG3	0.041	0.060	0.430	0.375	0.481	0.531	0.442	0.419	0.477	0.404
70150722	cg10801213	SLC7A3	0.026	0.033	0.418	0.336	0.487	0.537	0.456	0.480	0.484	0.531
70150787	cg11024449	SLC7A3	0.059	0.077	0.486	0.412	0.537	0.587	0.524	0.479	0.502	0.480
70338337	cg14961988	MED12	0.023	0.029	0.290	0.251	0.341	0.391	0.332	0.222	0.253	0.232
70338656	cg05511752	MED12	0.039	0.051	0.499	0.442	0.587	0.637	0.613	0.479	0.492	0.450
70473981	cg17603461	ZMYM3	0.023	0.044	0.380	0.294	0.441	0.491	0.403	0.313	0.359	0.398
70474085	cg26764972	ZMYM3	0.053	0.061	0.498	0.445	0.551	0.601	0.540	0.458	0.497	0.521
70474095	cg24770638	ZMYM3	0.051	0.066	0.478	0.445	0.526	0.576	0.533	0.481	0.441	0.559
70474110	cg21474854	ZMYM3	0.039	0.044	0.438	0.415	0.459	0.509	0.485	0.450	0.386	0.477
70474172	cg25226891	ZMYM3	0.042	0.059	0.405	0.346	0.493	0.543	0.517	0.414	0.386	0.369
70752659	cg09006810	OGT	0.029	0.048	0.339	0.289	0.415	0.465	0.354	0.337	0.433	0.274
70752808	cg16809091	OGT	0.029	0.037	0.351	0.282	0.417	0.467	0.390	0.323	0.432	0.349
71459039	cg07056550	ERCC6L	0.027	0.038	0.296	0.266	0.313	0.363	0.365	0.279	0.346	0.326
71459115	cg05279113	ERCC6L	0.036	0.048	0.471	0.430	0.528	0.578	0.513	0.450	0.458	0.460
71459119	cg12689022	ERCC6L	0.046	0.063	0.384	0.327	0.429	0.479	0.393	0.337	0.347	0.435
71793016	cg23472592	HDAC8	0.144	0.206	0.404	0.330	0.457	0.507	0.397	0.363	0.409	0.332
71793049	cg12234922	HDAC8	0.060	0.078	0.429	0.353	0.511	0.561	0.406	0.281	0.370	0.383
74144469	cg10078687	KIAA2022	0.067	0.074	0.447	0.419	0.472	0.522	0.461	0.468	0.456	0.473
74145215	cg15028497	KIAA2022	0.029	0.034	0.392	0.341	0.445	0.495	0.354	0.338	0.387	0.436
74376241	cg11340371	ABCB7	0.067	0.102	0.450	0.378	0.552	0.602	0.478	0.469	0.602	0.398
74376313	cg27419400	ABCB7	0.049	0.074	0.391	0.319	0.459	0.509	0.316	0.277	0.403	0.418
74743466	cg03353482	ZDHHC15	0.080	0.100	0.408	0.335	0.481	0.531	0.461	0.422	0.461	0.391
74743480	cg17509494	ZDHHC15	0.083	0.134	0.430	0.377	0.494	0.544	0.427	0.368	0.426	0.493
77041452	cg14061423	ATRX	0.076	0.089	0.375	0.286	0.436	0.486	0.306	0.343	0.391	0.299
77041745	cg12857702	ATRX	0.025	0.042	0.429	0.339	0.503	0.553	0.548	0.436	0.609	0.494
77041828	cg03661529	ATRX	0.030	0.042	0.465	0.393	0.512	0.562	0.494	0.445	0.525	0.472
77041832	cg00743068	ATRX	0.030	0.045	0.443	0.382	0.496	0.546	0.452	0.386	0.500	0.436
77041835	cg21889537	ATRX	0.018	0.032	0.334	0.275	0.435	0.485	0.315	0.341	0.283	0.391
77151319	cg02530767	MAGT1	0.044	0.071	0.496	0.421	0.601	0.651	0.555	0.471	0.510	0.522
77151529	cg23241240	MAGT1	0.081	0.121	0.491	0.428	0.570	0.620	0.550	0.450	0.679	0.484
83443097	cg26511293	RPS6KA6	0.066	0.094	0.341	0.302	0.421	0.471	0.341	0.285	0.379	0.421
83443104	cg08642787	RPS6KA6	0.051	0.062	0.417	0.365	0.464	0.514	0.489	0.416	0.539	0.469
99986436	cg27260927	SYTL4	0.091	0.116	0.422	0.362	0.448	0.498	**0.519**	0.442	0.456	0.488
99986526	cg17474263	SYTL4	0.043	0.058	0.400	0.350	0.445	0.495	**0.512**	0.367	0.377	0.336
100877773	cg09251061	ARMCX3	0.043	0.056	0.333	0.279	0.418	0.468	0.384	0.322	0.478	0.397
100878107	cg06900776	ARMCX3	0.041	0.053	0.324	0.268	0.371	0.421	0.385	0.362	0.354	0.290
100878134	cg12691219	ARMCX3	0.054	0.070	0.405	0.338	0.481	0.531	0.440	0.336	0.423	0.354
100878294	cg05832562	ARMCX3	0.015	0.021	0.355	0.290	0.435	0.485	0.369	0.309	0.376	0.310
100878326	cg11704309	ARMCX3	0.029	0.040	0.360	0.260	0.443	0.493	0.489	0.300	0.400	0.359
101380437	cg26937423	TCEAL2	0.028	0.044	0.375	0.341	0.405	0.455	0.422	0.313	0.375	0.302
101380592	cg14711543	TCEAL2	0.048	0.065	0.369	0.293	0.452	0.502	0.450	0.364	0.385	0.526
101380608	cg00611101	TCEAL2	0.030	0.043	0.341	0.265	0.416	0.466	0.370	0.290	0.289	0.425

101906537	cg10670396	GPRASP1	0.017	0.025	0.313	0.256	0.412	0.462	0.317	0.247	0.318	0.386
101906886	cg12295100	GPRASP1	0.071	0.093	0.407	0.347	0.475	0.525	0.378	0.405	0.491	0.349
103401777	cg03978312	MCART6	0.146	0.176	0.449	0.376	0.503	0.553	0.485	0.511	0.428	0.434
103401960	cg25367647	MCART6	0.020	0.028	0.441	0.393	0.513	0.563	0.432	0.458	0.488	0.353
106449802	cg26555756	CXorf41	0.045	0.063	0.400	0.334	0.447	0.497	0.436	0.369	0.364	0.256
106449854	cg17337467	CXorf41	0.060	0.087	0.371	0.336	0.420	0.470	0.455	0.339	0.358	0.368
106449867	cg06350796	CXorf41	0.020	0.041	0.355	0.269	0.424	0.474	0.497	0.408	0.433	0.429
106959829	cg24318558	TSC22D3	0.035	0.050	0.448	0.381	0.491	0.541	0.532	0.441	0.440	0.345
106959895	cg11907074	TSC22D3	0.038	0.048	0.337	0.307	0.387	0.437	0.460	0.317	0.328	0.351
107019244	cg03422380	TSC22D3	0.027	0.039	0.390	0.293	0.449	0.499	0.510	0.374	0.398	0.372
107019262	cg18677034	TSC22D3	0.033	0.037	0.449	0.405	0.520	0.570	0.513	0.446	0.433	0.411
107019333	cg23264750	TSC22D3	0.031	0.038	0.469	0.390	0.524	0.574	0.539	0.401	0.458	0.460
107019610	cg21456313	TSC22D3	0.024	0.034	0.499	0.396	0.609	0.659	0.615	0.478	0.488	0.555
107069001	cg15062055	MID2	0.052	0.072	0.340	0.255	0.412	0.462	0.428	0.400	0.287	0.392
107069417	cg19771541	MID2	0.060	0.080	0.408	0.351	0.444	0.494	0.511	0.477	0.370	0.460
107334275	cg27584800	ATG4A	0.038	0.066	0.426	0.373	0.496	0.546	0.620	0.478	0.546	0.547
107334626	cg18044482	ATG4A	0.018	0.022	0.362	0.327	0.403	0.453	0.424	0.346	0.351	0.378
107979988	cg00538420	IRS4	0.057	0.091	0.365	0.255	0.466	0.516	0.450	0.283	0.407	0.433
107980144	cg18161489	IRS4	0.132	0.190	0.350	0.287	0.404	0.454	0.420	0.371	0.370	0.401
109561508	cg13208429	AMMECR1	0.042	0.055	0.417	0.307	0.516	0.566	0.612	0.416	0.412	0.410
109561515	cg17959735	AMMECR1	0.041	0.049	0.392	0.324	0.463	0.513	0.503	0.340	0.374	0.369
109561747	cg05782651	AMMECR1	0.030	0.051	0.376	0.322	0.472	0.522	0.415	0.414	0.375	0.485
110038886	cg05921207	CHRDL1	0.087	0.115	0.469	0.411	0.521	0.571	0.560	0.476	0.472	0.481
110039078	cg19053358	CHRDL1	0.048	0.069	0.403	0.307	0.465	0.515	0.549	0.442	0.456	0.393
117250753	cg13024202	KLHL13	0.039	0.056	0.433	0.384	0.489	0.539	0.496	0.463	0.466	0.489
117250943	cg08447449	KLHL13	0.030	0.036	0.323	0.283	0.371	0.421	0.424	0.349	0.288	0.343
117250962	cg04029664	KLHL13	0.061	0.078	0.470	0.415	0.525	0.575	0.606	0.463	0.450	0.467
117251000	cg20786418	KLHL13	0.075	0.118	0.422	0.355	0.464	0.514	0.484	0.469	0.395	0.493
117480246	cg24597825	WDR44	0.027	0.038	0.447	0.405	0.482	0.532	0.503	0.459	0.466	0.434
117480893	cg27557909	WDR44	0.050	0.072	0.340	0.255	0.397	0.447	0.509	0.367	0.451	0.301
117630362	cg16586165	DOCK11	0.035	0.061	0.345	0.277	0.444	0.494	0.400	0.377	0.258	0.434
117630565	cg19334385	DOCK11	0.051	0.075	0.421	0.380	0.476	0.526	0.441	0.414	0.486	0.482
118370356	cg07876586	PGRMC1	0.031	0.043	0.377	0.325	0.459	0.509	0.389	0.439	0.385	0.351
118370461	cg05418443	PGRMC1	0.023	0.029	0.399	0.330	0.430	0.480	0.433	0.404	0.435	0.421
118533106	cg07256221	SLC25A43	0.039	0.043	0.475	0.447	0.507	0.557	0.456	0.427	0.478	0.400
118533786	cg04602218	SLC25A43	0.040	0.060	0.346	0.287	0.385	0.435	0.333	0.399	0.442	0.352
118739824	cg08214610	NKRF	0.018	0.023	0.351	0.304	0.418	0.468	0.333	0.308	0.396	0.417
118740132	cg23446945	NKRF	0.026	0.034	0.431	0.354	0.502	0.552	0.423	0.467	0.462	0.415
118826802	cg15845873	SEPT6	0.028	0.034	0.361	0.287	0.440	0.490	0.065	0.272	0.287	0.343
118826918	cg12393253	SEPT6	0.062	0.084	0.312	0.251	0.415	0.465	0.151	0.317	0.285	0.352
118827095	cg25553791	SEPT6	0.042	0.051	0.383	0.275	0.453	0.503	0.111	0.368	0.299	0.370
118986976	cg12527112	UPF3B	0.026	0.035	0.482	0.345	0.579	0.629	0.106	0.429	0.428	0.351
118987146	cg04184316	UPF3B	0.135	0.160	0.414	0.336	0.507	0.557	0.161	0.321	0.324	0.336
119077663	cg19410841	NKAP	0.031	0.040	0.467	0.408	0.628	0.678	**0.099**	0.375	0.462	0.426
119077820	cg03122511	NKAP	0.055	0.083	0.490	0.421	0.571	0.621	**0.105**	0.366	0.470	0.460
119078095	cg13475427	NKAP	0.102	0.119	0.457	0.402	0.557	0.607	0.154	0.442	0.406	0.456
119695102	cg25698940	CUL4B	0.030	0.043	0.353	0.306	0.404	0.454	**0.091**	0.343	0.338	0.315
119695210	cg08784874	CUL4B	0.025	0.036	0.492	0.442	0.557	0.607	**0.066**	0.383	0.411	0.397

119737620	cg12047536	MCTS1	0.035	0.055	0.404	0.317	0.476	0.526	**0.082**	0.318	0.349	0.379
119737675	cg05782751	MCTS1	0.081	0.126	0.448	0.330	0.543	0.593	**0.122**	0.462	0.501	0.479
119737885	cg25622910	MCTS1	0.034	0.064	0.300	0.253	0.355	0.405	**0.085**	0.272	0.311	0.298
119737891	cg24931094	MCTS1	0.056	0.077	0.350	0.278	0.411	0.461	**0.089**	0.280	0.337	0.316
119764206	cg07210774	C1GALT1C1	0.023	0.039	0.500	0.399	0.575	0.625	**0.085**	0.456	0.507	0.439
119764273	cg09238847	C1GALT1C1	0.037	0.046	0.298	0.257	0.353	0.403	**0.071**	0.250	0.264	0.242
122993037	cg23933262	XIAP	0.140	0.220	0.384	0.320	0.475	0.525	0.218	0.347	0.285	0.329
122993419	cg00920960	XIAP	0.018	0.028	0.394	0.355	0.461	0.511	**0.078**	0.322	0.358	0.382
122993679	cg06772067	XIAP	0.019	0.027	0.432	0.351	0.499	0.549	**0.086**	0.377	0.403	0.381
122993913	cg07874334	XIAP	0.029	0.040	0.440	0.351	0.527	0.577	**0.080**	0.386	0.441	0.387
122993976	cg17033281	XIAP	0.033	0.039	0.384	0.287	0.452	0.502	**0.068**	0.345	0.352	0.343
122994030	cg21711683	XIAP	0.020	0.026	0.411	0.356	0.468	0.518	**0.074**	0.352	0.407	0.409
122994069	cg09950034	XIAP	0.055	0.074	0.442	0.386	0.491	0.541	**0.134**	0.368	0.447	0.438
128674366	cg16716035	OCRL	0.033	0.044	0.399	0.276	0.498	0.548	**0.069**	0.356	0.345	0.388
128674686	cg13532816	OCRL	0.031	0.041	0.469	0.356	0.571	0.621	**0.090**	0.400	0.434	0.459
128913684	cg02283402	SASH3	0.025	0.036	0.382	0.333	0.463	0.513	**0.089**	0.335	0.400	0.343
128913786	cg10649042	SASH3	0.016	0.026	0.430	0.378	0.536	0.586	**0.076**	0.386	0.437	0.348
128913854	cg05872215	SASH3	0.033	0.044	0.415	0.362	0.510	0.560	**0.094**	0.331	0.356	0.313
129243806	cg21489390	ELF4	0.014	0.023	0.461	0.370	0.514	0.564	**0.047**	0.413	0.428	0.403
129243872	cg06428055	ELF4	0.024	0.037	0.426	0.362	0.481	0.531	**0.082**	0.325	0.377	0.404
129299924	cg05045028	AIFM1	0.033	0.048	0.385	0.309	0.447	0.497	**0.083**	0.334	0.370	0.353
129299948	cg06252876	AIFM1	0.024	0.031	0.378	0.309	0.452	0.502	**0.059**	0.326	0.348	0.335
129300230	cg01913024	AIFM1	0.038	0.046	0.414	0.339	0.514	0.564	**0.061**	0.314	0.386	0.350
129473599	cg24721916	SLC25A14	0.025	0.039	0.429	0.331	0.542	0.592	**0.056**	0.377	0.338	0.315
129473637	cg01108554	SLC25A14	0.030	0.053	0.454	0.358	0.543	0.593	**0.096**	0.410	0.470	0.398
129473931	cg23220418	SLC25A14	0.035	0.044	0.414	0.311	0.546	0.596	**0.070**	0.353	0.361	0.320
129474007	cg06734027	SLC25A14	0.056	0.074	0.454	0.422	0.521	0.571	**0.148**	0.399	0.446	0.439
130037175	cg01372992	ENOX2	0.032	0.042	0.347	0.265	0.420	0.470	**0.113**	0.335	0.316	0.358
130037457	cg17086681	ENOX2	0.030	0.036	0.425	0.297	0.529	0.579	**0.116**	0.399	0.454	0.387
130037593	cg06288403	ENOX2	0.029	0.052	0.447	0.336	0.527	0.577	**0.065**	0.319	0.317	0.341
130423130	cg01010618	IGSF1	0.038	0.064	0.371	0.280	0.432	0.482	**0.130**	0.440	0.400	0.456
130423289	cg04882894	IGSF1	0.050	0.086	0.400	0.325	0.468	0.518	**0.137**	0.348	0.364	0.443
130423431	cg18452672	IGSF1	0.146	0.173	0.319	0.254	0.360	0.410	0.217	0.334	0.331	0.300
131157326	cg00739787	MST4	0.031	0.050	0.334	0.290	0.398	0.448	**0.075**	0.380	0.303	0.319
131157336	cg25395546	MST4	0.021	0.030	0.360	0.257	0.459	0.509	**0.052**	0.302	0.252	0.300
131623259	cg21471515	MBNL3	0.033	0.046	0.333	0.283	0.414	0.464	**0.107**	0.336	0.419	0.361
131623878	cg18878700	MBNL3	0.028	0.036	0.416	0.290	0.481	0.531	**0.067**	0.349	0.339	0.354
131624026	cg12942133	MBNL3	0.020	0.044	0.489	0.424	0.546	0.596	**0.072**	0.419	0.467	0.466
132549625	cg10720577	GPC4	0.060	0.071	0.401	0.311	0.490	0.540	**0.113**	0.400	0.401	0.411
132550131	cg12600766	GPC4	0.051	0.075	0.437	0.329	0.551	0.601	**0.083**	0.380	0.304	0.347
133594210	cg19022966	HPRT1	0.082	0.108	0.496	0.417	0.596	0.646	**0.148**	0.423	0.456	0.427
133594951	cg02264284	HPRT1	0.019	0.032	0.380	0.280	0.472	0.522	**0.050**	0.276	0.334	0.292
133941107	cg14972002	FAM122C	0.025	0.034	0.342	0.273	0.399	0.449	**0.060**	0.314	0.309	0.281
133941121	cg12263679	FAM122C	0.012	0.022	0.338	0.294	0.407	0.457	**0.036**	0.318	0.339	0.301
133941190	cg24248049	FAM122C	0.032	0.049	0.369	0.286	0.422	0.472	**0.121**	0.314	0.393	0.377
133941225	cg01309671	FAM122C	0.044	0.055	0.382	0.286	0.473	0.523	**0.104**	0.312	0.341	0.269
133941745	cg15904427	FAM122C	0.016	0.025	0.397	0.306	0.477	0.527	**0.037**	0.365	0.401	0.383
134166046	cg01099541	FAM127A	0.032	0.040	0.384	0.305	0.456	0.506	**0.107**	0.336	0.382	0.322

134166205	cg15043283	FAM127A	0.049	0.068	0.485	0.335	0.584	0.634	**0.059**	0.350	0.377	0.372
134478785	cg14029201	ZNF75D	0.047	0.059	0.391	0.313	0.481	0.531	**0.093**	0.344	0.350	0.376
134478884	cg05908188	ZNF75D	0.025	0.035	0.438	0.350	0.491	0.541	**0.075**	0.369	0.402	0.371
135067468	cg22166305	SLC9A6	0.029	0.044	0.402	0.332	0.487	0.537	**0.066**	0.269	0.284	0.278
135068355	cg19860691	SLC9A6	0.038	0.069	0.492	0.417	0.570	0.620	**0.094**	0.459	0.528	0.494
135332975	cg08860608	MAP7D3	0.031	0.041	0.470	0.390	0.580	0.630	**0.111**	0.410	0.468	0.431
135333567	cg27047283	MAP7D3	0.026	0.036	0.490	0.419	0.543	0.593	**0.062**	0.468	0.482	0.475
135333705	cg13818628	MAP7D3	0.017	0.031	0.424	0.300	0.506	0.556	**0.084**	0.404	0.449	0.356
135333917	cg14601312	MAP7D3	0.043	0.067	0.398	0.334	0.487	0.537	**0.102**	0.354	0.450	0.421
135579980	cg19949137	HTATSF1	0.022	0.031	0.498	0.402	0.591	0.641	**0.084**	0.453	0.482	0.461
135580098	cg02698615	HTATSF1	0.028	0.042	0.409	0.319	0.485	0.535	**0.095**	0.362	0.364	0.406
135863323	cg09517019	ARHGEF6	0.046	0.067	0.443	0.319	0.563	0.613	**0.093**	0.340	0.422	0.519
135863582	cg01134144	ARHGEF6	0.110	0.147	0.352	0.292	0.467	0.517	**0.143**	0.232	0.263	0.185
136113882	cg13937075	GPR101	0.067	0.084	0.496	0.447	0.551	0.601	0.173	0.481	0.495	0.468
136113998	cg06223806	GPR101	0.041	0.051	0.375	0.311	0.467	0.517	**0.125**	0.296	0.311	0.356
136114047	cg05566296	GPR101	0.050	0.075	0.484	0.425	0.565	0.615	**0.113**	0.430	0.442	0.462
136114207	cg01228667	GPR101	0.024	0.040	0.346	0.286	0.417	0.467	**0.067**	0.302	0.329	0.277
139587184	cg02847500	SOX3	0.087	0.138	0.442	0.338	0.505	0.555	0.154	0.416	0.403	0.499
139587868	cg18496053	SOX3	0.142	0.211	0.424	0.344	0.514	0.564	0.214	0.397	0.459	0.509
140271259	cg09112156	LDOC1	0.049	0.073	0.492	0.442	0.535	0.585	**0.105**	0.422	0.425	0.458
140271314	cg11005845	LDOC1	0.058	0.070	0.396	0.266	0.521	0.571	**0.078**	0.317	0.354	0.306
142721811	cg10288278	SLITRK4	0.097	0.153	0.474	0.391	0.518	0.568	0.204	0.454	0.429	0.461
142721819	cg23297872	SLITRK4	0.073	0.135	0.426	0.338	0.522	0.572	0.177	0.399	0.368	0.439
144899567	cg25062147	SLITRK2	0.059	0.118	0.428	0.382	0.468	0.518	0.168	0.434	0.432	0.421
144899592	cg09610569	SLITRK2	0.058	0.103	0.470	0.405	0.527	0.577	0.159	0.451	0.461	0.446
148586734	cg02264182	IDS	0.059	0.088	0.499	0.436	0.592	0.642	**0.130**	0.472	0.478	0.494
148586931	cg09018810	IDS	0.032	0.053	0.464	0.421	0.530	0.580	**0.094**	0.409	0.420	0.406
148586978	cg17920241	IDS	0.031	0.045	0.436	0.351	0.486	0.536	**0.108**	0.450	0.418	0.431
148586983	cg17337021	IDS	0.033	0.042	0.498	0.422	0.603	0.653	**0.092**	0.435	0.422	0.466
149106647	cg09610589	LOC100272228	0.031	0.050	0.464	0.398	0.521	0.571	**0.087**	0.438	0.461	0.436
149106889	cg27327591	LOC100272228	0.039	0.049	0.409	0.302	0.461	0.511	**0.098**	0.391	0.408	0.379
151806225	cg00529027	GABRQ	0.074	0.101	0.450	0.358	0.532	0.582	0.158	0.403	0.424	0.389
151806352	cg18769303	GABRQ	0.061	0.081	0.476	0.406	0.526	0.576	0.166	0.461	0.454	0.476
152735637	cg07792041	HAUS7	0.032	0.044	0.333	0.264	0.444	0.494	**0.080**	0.315	0.315	0.330
152735785	cg24964466	HAUS7	0.025	0.041	0.454	0.364	0.536	0.586	**0.078**	0.434	0.414	0.428
152938907	cg25323012	PNCK	0.046	0.074	0.450	0.330	0.536	0.586	**0.123**	0.335	0.415	0.489
152938924	cg12919379	PNCK	0.076	0.107	0.417	0.343	0.479	0.529	**0.117**	0.348	0.362	0.378
152989592	cg09594760	BCAP31	0.073	0.096	0.467	0.380	0.580	0.630	**0.087**	0.315	0.395	0.390
152989895	cg04024675	BCAP31	0.039	0.051	0.478	0.379	0.567	0.617	**0.069**	0.337	0.377	0.388
152989902	cg11906884	BCAP31	0.050	0.071	0.391	0.314	0.482	0.532	**0.073**	0.279	0.295	0.301
152990043	cg06018273	BCAP31	0.030	0.039	0.492	0.358	0.575	0.625	**0.108**	0.408	0.441	0.450
153060166	cg14783837	IDH3G	0.042	0.062	0.338	0.266	0.414	0.464	**0.070**	0.311	0.338	0.342
153060354	cg26105341	IDH3G	0.072	0.098	0.442	0.309	0.548	0.598	**0.058**	0.315	0.378	0.369
153602658	cg03672021	FLNA	0.046	0.075	0.450	0.378	0.506	0.556	**0.141**	0.410	0.446	0.462
153603126	cg15879692	FLNA	0.043	0.065	0.346	0.270	0.408	0.458	**0.095**	0.355	0.429	0.348
153640960	cg16405946	DNASE1L1	0.036	0.044	0.498	0.437	0.575	0.625	**0.109**	0.427	0.460	0.460
153640967	cg05100268	DNASE1L1	0.028	0.047	0.480	0.377	0.554	0.604	**0.084**	0.401	0.497	0.445
153672393	cg08408091	FAM50A	0.048	0.072	0.473	0.369	0.555	0.605	**0.068**	0.348	0.419	0.389

153672689	cg20616045	FAM50A	0.026	0.033	0.412	0.343	0.483	0.533	**0.055**	0.330	0.378	0.382
153775885	cg00813156	IKBKG	0.035	0.047	0.462	0.366	0.541	0.591	**0.054**	0.381	0.401	0.397
153775995	cg13550079	IKBKG	0.037	0.060	0.448	0.324	0.547	0.597	**0.116**	0.387	0.379	0.417
153979491	cg10026671	GAB3	0.081	0.111	0.424	0.379	0.504	0.554	**0.112**	0.436	0.382	0.306
153979543	cg23896806	GAB3	0.049	0.074	0.395	0.348	0.487	0.537	**0.120**	0.324	0.334	0.366
154254936	cg03172171	FUNDC2	0.051	0.113	0.411	0.369	0.486	0.536	**0.104**	0.371	0.441	0.422
154254947	cg26116400	FUNDC2	0.038	0.070	0.383	0.317	0.451	0.501	**0.070**	0.313	0.393	0.366
154255534	cg21697779	FUNDC2	0.028	0.052	0.416	0.324	0.524	0.574	**0.089**	0.358	0.428	0.358

A yellow background means probes in duplicated regions. A pale blue background means probes in deleted regions. When the βvalue is higher than 0.5 and the βvalue is higher than the maximum level of female controls + 0.05, the βvalue is shown in red letters. When more than two consecutive probes' βvalue within 1,000 bp from TSS of a gene satisfy this criteria, the βvalue is shown in red bold letters. The β value less than 0.15 is shown in blue letters. When more than two consecutive probes' βvalue within 1,000 bp from TSS of a gene satisfy this criteria, the βvalue is shown in blue bold letters.
