# Supplementary material for: Methylation status of genes escaping from X-chromosome inactivation in patients with X-chromosome rearrangements
Source: Clin Epigenetics. 2021 Jun 30;13:134. doi: 10.1186/s13148-021-01121-6 (PMC8244138; doi:10.1186/s13148-021-01121-6)
Supplement: Supplementary file 8 — Additional file 8: Table S5. The methylation ratio of CpGs in promoter regions of genes in PAR1. [file 13148_2021_1121_MOESM8_ESM.pdf]

Table S5. The methylation ratio of CpGs in promoter regions of genes in PAR1

Genomic position	Gene name	Female control Mean methylation ratio	Patient 1 methylation ratio	Patient 2 methylation ratio	Patient 3 methylation ratio	Patient 4 methylation ratio	Coverage Patient 1	Coverage Patient 2	Coverage Patient 3	Coverage Patient 4
192148	PLCXD1	0.13	0.10	0.07	0.00	0.00	21	45	20	19
192149	PLCXD1	0.02	0.00	0.00	0.00	0.00	11	21	16	15
192327	PLCXD1	0.00	0.20	0.09	0.00	0.00	10	11	16	11
192430	PLCXD1	0.11	0.40	0.09	0.00	0.00	10	11	16	11
192433	PLCXD1	0.08	0.20	0.09	0.00	0.00	10	11	16	11
192460	PLCXD1	0.10	0.20	0.09	0.00	0.09	10	11	16	11
192631	PLCXD1	0.00	0.09	0.00	0.00	0.00	11	18	25	11
192637	PLCXD1	0.02	0.09	0.00	0.00	0.00	11	18	25	11
192639	PLCXD1	0.01	0.18	0.00	0.00	0.00	11	17	25	11
192651	PLCXD1	0.02	0.00	0.00	0.00	0.00	11	18	25	11
192662	PLCXD1	0.00	0.09	0.00	0.00	0.00	11	18	25	11
192717	PLCXD1	0.01	0.00	0.00	0.00	0.00	11	19	24	11
192738	PLCXD1	0.00	0.00	0.00	0.00	0.00	11	19	24	11
192765	PLCXD1	0.06	0.18	0.00	0.00	0.00	11	19	24	10
193646	PLCXD1	0.01	0.00	0.00	0.00	0.00	10	22	30	12
193656	PLCXD1	0.00	0.00	0.05	0.10	0.00	10	22	30	12
193660	PLCXD1	0.03	0.00	0.05	0.10	0.08	10	22	30	12
193669	PLCXD1	0.08	0.00	0.05	0.03	0.00	10	21	30	12
193677	PLCXD1	0.05	0.00	0.05	0.00	0.08	10	22	30	12
193680	PLCXD1	0.01	0.00	0.00	0.03	0.00	10	22	30	12
193682	PLCXD1	0.01	0.00	0.00	0.00	0.00	10	22	30	12
193686	PLCXD1	0.04	0.00	0.00	0.00	0.00	10	22	30	12
193734	PLCXD1	0.03	0.00	0.05	0.03	0.00	10	22	30	12
193741	PLCXD1	0.00	0.00	0.09	0.07	0.00	10	22	30	12
193760	PLCXD1	0.05	0.00	0.00	0.17	0.00	10	22	30	12
193768	PLCXD1	0.01	0.00	0.00	0.03	0.00	10	22	30	12
347497	PPP2R3B	0.08	0.00	0.04	0.06	0.00	15	24	33	13
347502	PPP2R3B	0.00	0.00	0.00	0.00	0.00	15	24	33	13
347508	PPP2R3B	0.00	0.00	0.00	0.00	0.00	15	24	33	13
347516	PPP2R3B	0.00	0.00	0.04	0.00	0.00	15	24	33	13
347522	PPP2R3B	0.00	0.00	0.00	0.00	0.00	15	24	33	13
347527	PPP2R3B	0.00	0.00	0.00	0.00	0.00	15	24	33	13

347538	PPP2R3B	0.01	0.00	0.00	0.03	0.00	15	24	33	13
347540	PPP2R3B	0.01	0.00	0.04	0.00	0.00	15	24	33	13
347543	PPP2R3B	0.00	0.00	0.04	0.00	0.00	15	24	33	13
347559	PPP2R3B	0.01	0.00	0.00	0.03	0.00	15	24	33	13
347561	PPP2R3B	0.00	0.00	0.00	0.00	0.00	15	24	33	13
347566	PPP2R3B	0.00	0.00	0.00	0.00	0.00	15	24	33	13
347575	PPP2R3B	0.01	0.07	0.04	0.00	0.00	15	24	33	13
347577	PPP2R3B	0.00	0.00	0.00	0.00	0.00	15	24	33	13
347583	PPP2R3B	0.00	0.00	0.00	0.03	0.00	15	24	33	13
347585	PPP2R3B	0.05	0.00	0.00	0.03	0.00	15	24	33	13
347590	PPP2R3B	0.02	0.00	0.04	0.00	0.00	15	24	33	13
347592	PPP2R3B	0.00	0.00	0.00	0.00	0.00	15	24	33	13
347599	PPP2R3B	0.01	0.00	0.00	0.00	0.00	15	24	33	13
347610	PPP2R3B	0.00	0.00	0.00	0.00	0.00	15	24	32	13
347612	PPP2R3B	0.01	0.00	0.00	0.00	0.00	15	24	32	13
348019	PPP2R3B	0.08	0.09	0.00	0.00	0.09	23	23	27	11
348023	PPP2R3B	0.00	0.00	0.00	0.00	0.00	21	19	26	11
348024	PPP2R3B	0.00	0.00	0.00	0.00	0.00	14	20	10	10
348031	PPP2R3B	0.00	0.00	0.00	0.00	0.00	21	19	25	11
348032	PPP2R3B	0.00	0.00	0.00	0.00	0.00	14	20	10	11
348035	PPP2R3B	0.00	0.00	0.00	0.00	0.00	21	19	26	11
348036	PPP2R3B	0.00	0.00	0.00	0.00	0.00	14	20	10	11
348048	PPP2R3B	0.00	0.00	0.05	0.00	0.00	21	19	27	11
348049	PPP2R3B	0.00	0.00	0.00	0.00	0.00	14	20	10	11
348050	PPP2R3B	0.00	0.00	0.00	0.00	0.00	21	19	27	11
348051	PPP2R3B	0.01	0.00	0.00	0.00	0.00	14	20	10	11
348055	PPP2R3B	0.00	0.00	0.00	0.00	0.00	21	19	27	11
348056	PPP2R3B	0.00	0.00	0.00	0.00	0.00	14	20	10	11
348084	PPP2R3B	0.01	0.00	0.00	0.00	0.00	21	19	27	11
348085	PPP2R3B	0.01	0.00	0.00	0.00	0.00	14	20	10	11
348101	PPP2R3B	0.00	0.05	0.00	0.00	0.00	21	19	27	11
348102	PPP2R3B	0.00	0.00	0.00	0.00	0.00	14	20	10	11
348109	PPP2R3B	0.00	0.00	0.05	0.00	0.00	21	19	27	11
348110	PPP2R3B	0.00	0.07	0.00	0.00	0.00	14	20	10	11
348115	PPP2R3B	0.01	0.00	0.00	0.00	0.00	21	19	27	11
348116	PPP2R3B	0.00	0.00	0.00	0.00	0.00	14	20	10	11

348131	PPP2R3B	0.06	0.00	0.00	0.10	0.00	15	19	10	10
584580	SHOX	0.09	0.00	0.05	0.00	0.00	18	21	25	10
584590	SHOX	0.06	0.00	0.10	0.00	0.30	18	20	26	10
584591	SHOX	0.02	0.00	0.00	0.00	0.00	20	38	39	16
584592	SHOX	0.04	0.00	0.05	0.00	0.00	18	20	26	10
584593	SHOX	0.06	0.00	0.00	0.05	0.00	20	38	39	16
584613	SHOX	0.02	0.00	0.05	0.04	0.00	18	20	26	10
584614	SHOX	0.02	0.05	0.03	0.03	0.00	20	38	39	16
584636	SHOX	0.01	0.11	0.05	0.00	0.10	18	20	26	10
584637	SHOX	0.02	0.00	0.03	0.00	0.00	20	38	39	16
584642	SHOX	0.01	0.00	0.10	0.00	0.00	18	20	26	10
584643	SHOX	0.01	0.00	0.03	0.05	0.00	20	38	39	16
584654	SHOX	0.01	0.00	0.05	0.00	0.00	18	20	26	10
584655	SHOX	0.03	0.00	0.03	0.03	0.00	20	38	39	16
584682	SHOX	0.01	0.00	0.05	0.04	0.00	18	20	26	10
584683	SHOX	0.01	0.00	0.00	0.00	0.06	20	38	39	16
584693	SHOX	0.00	0.00	0.05	0.00	0.00	18	20	26	10
584694	SHOX	0.01	0.00	0.00	0.03	0.00	20	38	39	16
584703	SHOX	0.05	0.00	0.18	0.17	0.14	26	39	12	14
584704	SHOX	0.03	0.08	0.02	0.00	0.11	25	47	47	18
1510466	SLC25A6	0.05	0.00	0.06	0.00	0.03	13	18	17	35
1510475	SLC25A6	0.00	0.00	0.00	0.00	0.00	13	18	19	35
1510476	SLC25A6	0.00	0.00	0.09	0.00	0.00	19	23	16	32
1510502	SLC25A6	0.01	0.00	0.00	0.05	0.00	13	18	19	35
1510503	SLC25A6	0.01	0.00	0.00	0.00	0.00	20	23	16	32
1510509	SLC25A6	0.02	0.00	0.00	0.00	0.00	13	18	19	35
1510510	SLC25A6	0.01	0.00	0.00	0.00	0.00	20	23	16	32
1510518	SLC25A6	0.00	0.00	0.00	0.00	0.00	13	18	19	35
1510519	SLC25A6	0.00	0.00	0.00	0.00	0.00	20	23	16	32
1510538	SLC25A6	0.00	0.00	0.00	0.00	0.00	13	18	19	35
1510539	SLC25A6	0.01	0.00	0.00	0.00	0.00	20	23	17	32
1510540	SLC25A6	0.00	0.00	0.00	0.00	0.00	13	18	19	35
1510541	SLC25A6	0.01	0.00	0.00	0.00	0.00	20	23	17	32
1510546	SLC25A6	0.01	0.00	0.00	0.00	0.00	13	18	19	35
1510547	SLC25A6	0.01	0.00	0.04	0.00	0.03	20	23	17	32
1510558	SLC25A6	0.00	0.00	0.00	0.00	0.00	13	18	19	35

1510559	SLC25A6	0.02	0.00	0.00	0.06	0.00	20	23	17	32
1510563	SLC25A6	0.00	0.00	0.00	0.00	0.00	13	18	19	35
1510564	SLC25A6	0.00	0.00	0.00	0.00	0.00	20	23	17	32
1510575	SLC25A6	0.01	0.00	0.00	0.00	0.00	13	18	19	35
1510576	SLC25A6	0.00	0.00	0.00	0.00	0.00	20	23	17	32
1510579	SLC25A6	0.00	0.00	0.00	0.00	0.00	13	18	19	35
1510580	SLC25A6	0.01	0.00	0.04	0.00	0.00	20	23	17	32
1510583	SLC25A6	0.04	0.04	0.03	0.00	0.00	23	32	20	35
1510766	SLC25A6	0.08	0.00	0.05	0.04	0.05	22	21	23	43
1510771	SLC25A6	0.02	0.00	0.00	0.00	0.00	23	22	25	44
1510772	SLC25A6	0.01	0.00	0.00	0.00	0.02	20	20	15	42
1510775	SLC25A6	0.01	0.00	0.00	0.00	0.00	23	22	25	44
1510776	SLC25A6	0.01	0.00	0.00	0.00	0.00	20	20	15	42
1510777	SLC25A6	0.01	0.00	0.00	0.00	0.00	23	22	25	44
1510778	SLC25A6	0.01	0.00	0.00	0.00	0.00	20	20	15	42
1510781	SLC25A6	0.01	0.00	0.00	0.04	0.00	23	22	25	44
1510782	SLC25A6	0.01	0.00	0.00	0.00	0.00	20	20	15	42
1510783	SLC25A6	0.00	0.00	0.00	0.00	0.00	22	22	25	44
1510784	SLC25A6	0.00	0.00	0.00	0.00	0.00	20	20	15	42
1510811	SLC25A6	0.00	0.00	0.00	0.00	0.02	23	22	25	44
1510812	SLC25A6	0.00	0.00	0.00	0.00	0.00	19	20	15	42
1510815	SLC25A6	0.00	0.04	0.00	0.00	0.02	23	22	25	44
1510816	SLC25A6	0.00	0.00	0.00	0.00	0.00	20	20	15	42
1510819	SLC25A6	0.01	0.00	0.00	0.04	0.00	23	22	25	44
1510820	SLC25A6	0.01	0.00	0.00	0.00	0.02	20	20	15	42
1510827	SLC25A6	0.00	0.00	0.00	0.00	0.00	23	22	25	44
1510828	SLC25A6	0.00	0.00	0.00	0.00	0.00	20	20	15	42
1510831	SLC25A6	0.02	0.00	0.00	0.00	0.00	23	22	25	44
1510832	SLC25A6	0.00	0.00	0.00	0.00	0.00	20	20	15	42
1510845	SLC25A6	0.01	0.00	0.00	0.04	0.00	23	22	25	44
1510846	SLC25A6	0.03	0.00	0.00	0.00	0.00	20	20	15	42
1510848	SLC25A6	0.00	0.00	0.00	0.00	0.02	23	22	25	44
1510849	SLC25A6	0.01	0.05	0.00	0.00	0.00	20	20	15	42
1510851	SLC25A6	0.01	0.00	0.00	0.04	0.00	23	22	25	44
1510852	SLC25A6	0.00	0.00	0.00	0.00	0.02	20	20	15	42
1510860	SLC25A6	0.03	0.08	0.00	0.10	0.00	24	25	31	47

1510861	SLC25A6	0.08	0.04	0.06	0.00	0.02	23	36	22	54
1510937	SLC25A6	0.00	0.00	0.00	0.04	0.00	14	20	27	32
1511148	SLC25A6	0.06	0.09	0.06	0.07	0.00	11	17	14	30
1511241	SLC25A6	0.02	0.06	0.00	0.00	0.00	17	16	29	23
1511255	SLC25A6	0.00	0.12	0.00	0.00	0.04	17	16	29	23
1511263	SLC25A6	0.00	0.06	0.00	0.00	0.00	17	16	29	23
1511266	SLC25A6	0.00	0.06	0.00	0.00	0.00	17	16	29	23
1511275	SLC25A6	0.01	0.06	0.00	0.03	0.04	17	16	29	23
1511287	SLC25A6	0.00	0.06	0.00	0.00	0.00	17	16	29	23
1511289	SLC25A6	0.00	0.06	0.00	0.00	0.00	17	16	29	23
1511318	SLC25A6	0.00	0.06	0.06	0.00	0.00	16	16	29	23
1511331	SLC25A6	0.00	0.00	0.06	0.00	0.00	16	16	29	23
1511335	SLC25A6	0.00	0.00	0.00	0.00	0.00	16	16	29	23
1511339	SLC25A6	0.00	0.00	0.00	0.00	0.00	16	16	29	23
1511353	SLC25A6	0.01	0.06	0.00	0.00	0.00	16	16	29	23
1511384	SLC25A6	0.00	0.00	0.00	0.00	0.00	16	16	29	23
1511389	SLC25A6	0.00	0.06	0.00	0.00	0.00	16	16	29	23
1511395	SLC25A6	0.00	0.06	0.00	0.00	0.00	16	16	29	23
1511398	SLC25A6	0.00	0.06	0.00	0.00	0.00	16	16	28	23
1511402	SLC25A6	0.00	0.06	0.00	0.00	0.00	16	16	29	23
1511558	SLC25A6	0.04	0.00	0.00	0.08	0.00	11	16	13	23
1511568	SLC25A6	0.00	0.00	0.00	0.00	0.00	11	16	14	23
1511569	SLC25A6	0.00	0.00	0.00	0.00	0.00	23	14	15	31
1511570	SLC25A6	0.00	0.00	0.00	0.07	0.04	11	16	14	23
1511571	SLC25A6	0.00	0.00	0.00	0.00	0.00	23	14	15	31
1511572	SLC25A6	0.00	0.00	0.00	0.00	0.00	11	16	14	23
1511573	SLC25A6	0.00	0.00	0.00	0.00	0.00	23	14	15	31
1511581	SLC25A6	0.00	0.00	0.00	0.00	0.00	11	16	14	23
1511582	SLC25A6	0.00	0.00	0.00	0.00	0.03	23	14	15	30
1511584	SLC25A6	0.00	0.00	0.00	0.00	0.00	11	16	14	23
1511585	SLC25A6	0.00	0.00	0.00	0.00	0.00	23	14	15	31
1511593	SLC25A6	0.00	0.00	0.00	0.00	0.00	11	16	14	23
1511594	SLC25A6	0.01	0.00	0.00	0.00	0.00	23	14	15	31
1511599	SLC25A6	0.00	0.00	0.00	0.00	0.00	11	16	14	23
1511600	SLC25A6	0.00	0.00	0.00	0.00	0.00	23	14	15	31
1511601	SLC25A6	0.00	0.00	0.00	0.00	0.00	11	16	14	22

1511602	SLC25A6	0.02	0.00	0.00	0.00	0.00	23	14	15	31
1511612	SLC25A6	0.00	0.00	0.00	0.00	0.00	11	16	14	23
1511613	SLC25A6	0.01	0.00	0.00	0.00	0.00	23	14	15	31
1511618	SLC25A6	0.00	0.00	0.00	0.00	0.00	11	16	14	23
1511619	SLC25A6	0.00	0.00	0.00	0.00	0.03	23	14	15	31
1511623	SLC25A6	0.00	0.00	0.00	0.00	0.04	11	16	14	23
1511624	SLC25A6	0.00	0.00	0.00	0.00	0.00	23	14	15	31
1511631	SLC25A6	0.03	0.00	0.00	0.00	0.00	11	16	14	23
1511632	SLC25A6	0.00	0.00	0.00	0.00	0.00	23	14	15	31
1511633	SLC25A6	0.00	0.00	0.06	0.00	0.00	11	16	14	23
1511634	SLC25A6	0.00	0.00	0.00	0.00	0.00	23	14	15	31
1511636	SLC25A6	0.00	0.00	0.00	0.00	0.00	11	16	13	23
1511637	SLC25A6	0.01	0.00	0.00	0.00	0.00	23	14	15	31
1511643	SLC25A6	0.04	0.00	0.00	0.00	0.00	11	16	14	23
1511644	SLC25A6	0.02	0.00	0.00	0.00	0.00	23	14	15	31
1511645	SLC25A6	0.00	0.00	0.00	0.00	0.00	11	16	14	23
1511646	SLC25A6	0.00	0.09	0.00	0.00	0.00	23	14	15	31
1511650	SLC25A6	0.02	0.00	0.00	0.07	0.00	24	17	15	33
1570938	ASMTL	0.11	0.11	0.16	0.05	0.10	37	43	38	90
1571536	ASMTL	0.00	0.00	0.03	0.00	0.00	33	37	17	53
1571575	ASMTL	0.00	0.00	0.00	0.00	0.02	33	37	17	53
1571587	ASMTL	0.13	0.06	0.03	0.00	0.08	33	37	16	53
1571608	ASMTL	0.09	0.06	0.00	0.09	0.13	17	27	22	23
1571621	ASMTL	0.00	0.00	0.00	0.00	0.00	17	27	22	25
1571622	ASMTL	0.00	0.00	0.00	0.00	0.00	17	21	20	37
1571636	ASMTL	0.01	0.00	0.04	0.05	0.00	17	27	22	25
1571637	ASMTL	0.00	0.00	0.00	0.00	0.00	17	21	20	37
1571641	ASMTL	0.03	0.00	0.00	0.00	0.00	17	27	22	25
1571642	ASMTL	0.00	0.00	0.00	0.00	0.03	17	21	20	37
1571643	ASMTL	0.00	0.00	0.00	0.00	0.00	17	27	22	25
1571644	ASMTL	0.00	0.00	0.00	0.00	0.00	17	21	20	37
1571663	ASMTL	0.00	0.00	0.00	0.00	0.00	17	27	22	25
1571664	ASMTL	0.01	0.00	0.00	0.00	0.03	17	21	20	37
1571666	ASMTL	0.00	0.00	0.00	0.00	0.00	17	27	22	25
1571667	ASMTL	0.00	0.00	0.00	0.00	0.00	17	21	20	37
1571676	ASMTL	0.00	0.00	0.00	0.00	0.00	17	27	22	25

1571677	ASMTL	0.00	0.00	0.00	0.00	0.00	17	21	20	37
1571691	ASMTL	0.00	0.00	0.00	0.00	0.00	17	27	22	25
1571692	ASMTL	0.01	0.00	0.00	0.00	0.00	17	21	20	37
1571693	ASMTL	0.01	0.00	0.00	0.00	0.00	17	27	22	25
1571694	ASMTL	0.00	0.00	0.00	0.00	0.03	17	21	19	37
1571720	ASMTL	0.08	0.18	0.00	0.00	0.03	17	21	20	37
1572529	ASMTL	0.03	0.00	0.00	0.00	0.00	10	20	11	21
1573211	ASMTL	0.02	0.00	0.00	0.00	0.03	12	18	30	39
1573232	ASMTL	0.00	0.00	0.00	0.00	0.03	12	18	30	39
1573243	ASMTL	0.01	0.00	0.00	0.00	0.00	12	18	30	39
1573245	ASMTL	0.00	0.00	0.00	0.03	0.03	12	18	30	39
1573248	ASMTL	0.00	0.00	0.00	0.00	0.03	12	18	30	39
1573264	ASMTL	0.00	0.00	0.00	0.00	0.03	12	18	29	39
1573266	ASMTL	0.00	0.00	0.00	0.00	0.03	12	18	30	39
1573268	ASMTL	0.00	0.00	0.00	0.00	0.03	12	18	30	40
1573279	ASMTL	0.02	0.00	0.00	0.00	0.03	12	18	30	40
1573310	ASMTL	0.00	0.00	0.00	0.00	0.03	12	18	30	40
1573334	ASMTL	0.01	0.00	0.00	0.00	0.03	12	18	29	40
1573339	ASMTL	0.00	0.00	0.00	0.04	0.03	12	18	28	40
1573350	ASMTL	0.05	0.08	0.06	0.00	0.10	12	18	27	40
1655910	P2RY8	0.04	0.00	0.00	0.00	0.02	17	11	23	43
1655917	P2RY8	0.00	0.00	0.00	0.00	0.00	17	12	25	44
1655918	P2RY8	0.01	0.00	0.00	0.00	0.00	15	26	32	37
1655944	P2RY8	0.00	0.00	0.00	0.00	0.00	17	12	25	44
1655945	P2RY8	0.01	0.00	0.00	0.00	0.00	15	26	32	37
1655975	P2RY8	0.00	0.00	0.00	0.04	0.00	17	12	26	45
1655976	P2RY8	0.01	0.00	0.00	0.00	0.00	15	26	32	37
1655981	P2RY8	0.00	0.00	0.00	0.00	0.00	17	11	26	45
1655982	P2RY8	0.00	0.00	0.00	0.03	0.00	15	26	32	37
1655988	P2RY8	0.01	0.00	0.00	0.00	0.00	17	12	26	45
1655989	P2RY8	0.00	0.00	0.00	0.00	0.00	15	25	32	37
1656031	P2RY8	0.00	0.00	0.00	0.00	0.00	17	12	26	45
1656032	P2RY8	0.02	0.00	0.00	0.00	0.00	15	26	32	37
1656035	P2RY8	0.00	0.00	0.00	0.00	0.00	17	12	26	45
1656036	P2RY8	0.01	0.00	0.00	0.00	0.00	15	26	31	37
1656040	P2RY8	0.00	0.00	0.00	0.00	0.00	17	11	26	45

1656041	P2RY8	0.01	0.07	0.00	0.00	0.00	15	26	32	37
1656056	P2RY8	0.03	0.00	0.00	0.06	0.00	14	26	33	38
1710331	AKAP17A	0.01	0.00	0.00	0.00	0.00	15	23	22	38
1710355	AKAP17A	0.00	0.00	0.00	0.00	0.00	15	23	21	38
1710360	AKAP17A	0.01	0.00	0.00	0.00	0.00	15	23	21	38
1710366	AKAP17A	0.00	0.00	0.00	0.00	0.00	15	23	21	38
1710368	AKAP17A	0.00	0.00	0.00	0.00	0.00	15	23	21	38
1710372	AKAP17A	0.00	0.00	0.00	0.00	0.03	15	23	21	38
1710374	AKAP17A	0.00	0.00	0.00	0.00	0.00	15	23	21	38
1710389	AKAP17A	0.01	0.00	0.00	0.00	0.00	15	23	21	38
1710397	AKAP17A	0.04	0.00	0.09	0.00	0.00	15	23	20	38
1710587	AKAP17A	0.05	0.00	0.00	0.00	0.07	11	18	18	29
1710594	AKAP17A	0.01	0.09	0.00	0.00	0.03	11	19	18	29
1710595	AKAP17A	0.00	0.00	0.00	0.00	0.00	13	26	15	45
1710597	AKAP17A	0.00	0.00	0.00	0.00	0.00	11	19	18	29
1710598	AKAP17A	0.00	0.00	0.00	0.00	0.00	13	26	15	45
1710604	AKAP17A	0.00	0.00	0.00	0.00	0.00	11	19	18	29
1710605	AKAP17A	0.01	0.00	0.00	0.00	0.00	13	26	15	45
1710607	AKAP17A	0.00	0.00	0.00	0.00	0.00	11	19	18	29
1710608	AKAP17A	0.00	0.00	0.00	0.00	0.00	13	25	15	45
1710623	AKAP17A	0.01	0.00	0.00	0.00	0.00	11	19	18	29
1710624	AKAP17A	0.00	0.00	0.00	0.00	0.00	13	26	15	45
1710626	AKAP17A	0.00	0.00	0.00	0.00	0.00	11	19	18	29
1710627	AKAP17A	0.00	0.00	0.04	0.07	0.00	13	26	15	45
1710629	AKAP17A	0.01	0.00	0.00	0.00	0.00	11	19	18	29
1710630	AKAP17A	0.01	0.00	0.00	0.07	0.02	13	26	15	45
1710632	AKAP17A	0.01	0.00	0.00	0.00	0.00	11	19	18	29
1710633	AKAP17A	0.01	0.00	0.00	0.00	0.02	13	26	15	45
1710642	AKAP17A	0.01	0.00	0.00	0.00	0.00	11	19	18	29
1710643	AKAP17A	0.00	0.00	0.00	0.00	0.00	13	26	15	45
1710648	AKAP17A	0.01	0.00	0.05	0.00	0.00	11	19	18	29
1710649	AKAP17A	0.00	0.00	0.00	0.00	0.00	13	26	15	45
1710656	AKAP17A	0.00	0.00	0.00	0.00	0.00	11	19	18	29
1710657	AKAP17A	0.00	0.00	0.00	0.00	0.00	13	26	15	45
1710658	AKAP17A	0.00	0.00	0.00	0.00	0.00	11	19	18	29
1710659	AKAP17A	0.00	0.00	0.00	0.00	0.00	13	26	15	45

1710671	AKAP17A	0.00	0.00	0.00	0.00	0.00	11	19	18	29
1710672	AKAP17A	0.00	0.00	0.00	0.00	0.00	13	26	15	45
1710677	AKAP17A	0.00	0.00	0.00	0.00	0.00	11	19	18	29
1710678	AKAP17A	0.01	0.00	0.00	0.00	0.02	13	26	15	45
1710687	AKAP17A	0.05	0.00	0.00	0.13	0.00	13	23	15	41
1710930	AKAP17A	0.01	0.00	0.00	0.00	0.00	11	10	20	15
1710951	AKAP17A	0.01	0.00	0.10	0.00	0.00	11	10	20	15
1710957	AKAP17A	0.01	0.00	0.00	0.05	0.00	11	10	21	15
1710973	AKAP17A	0.01	0.00	0.00	0.05	0.00	11	10	21	15
1710981	AKAP17A	0.01	0.00	0.00	0.05	0.14	11	10	21	14
1710989	AKAP17A	0.00	0.00	0.00	0.00	0.00	11	10	21	15
1711019	AKAP17A	0.00	0.00	0.00	0.00	0.00	11	10	21	14
1711024	AKAP17A	0.01	0.00	0.00	0.00	0.00	11	10	21	15
1711029	AKAP17A	0.01	0.09	0.00	0.00	0.07	11	10	21	15
1711033	AKAP17A	0.00	0.00	0.00	0.00	0.00	11	10	21	15
2527287	CD99P1	0.00	0.00	0.00	0.00	0.00	12	25	13	30
2527292	CD99P1	0.02	0.00	0.00	0.00	0.00	12	25	13	30
2527301	CD99P1	0.00	0.08	0.00	0.08	0.00	12	25	13	30
2527349	CD99P1	0.00	0.00	0.00	0.00	0.03	12	25	13	30
2527358	CD99P1	0.01	0.00	0.00	0.00	0.00	12	25	14	30
2527360	CD99P1	0.00	0.00	0.00	0.00	0.00	12	25	14	30
2527367	CD99P1	0.01	0.00	0.00	0.00	0.00	12	25	14	30
2527376	CD99P1	0.06	0.06	0.00	0.00	0.13	16	34	22	32
2527555	CD99P1	0.00	0.00	0.00	0.00	0.00	15	14	17	18
2527557	CD99P1	0.00	0.00	0.00	0.00	0.00	15	14	17	17
2527567	CD99P1	0.00	0.00	0.00	0.00	0.00	15	14	17	18
2527569	CD99P1	0.00	0.00	0.00	0.00	0.00	15	14	17	18
2527613	CD99P1	0.00	0.00	0.00	0.00	0.00	15	14	17	18
2527618	CD99P1	0.00	0.00	0.00	0.00	0.00	15	14	17	18
2527623	CD99P1	0.00	0.00	0.00	0.00	0.00	15	14	17	18
2527625	CD99P1	0.00	0.00	0.00	0.00	0.00	15	14	17	18
2527635	CD99P1	0.05	0.07	0.00	0.00	0.00	15	14	16	17
2527687	CD99P1	0.09	0.00	0.04	0.04	0.10	33	28	28	40
2527696	CD99P1	0.02	0.00	0.00	0.00	0.00	33	29	30	42
2527697	CD99P1	0.00	0.04	0.00	0.00	0.00	23	18	17	35
2527714	CD99P1	0.01	0.00	0.00	0.00	0.00	33	28	30	42

2527715	CD99P1	0.01	0.04	0.00	0.00	0.00	23	18	17	35
2527740	CD99P1	0.01	0.00	0.00	0.03	0.00	33	29	30	42
2527741	CD99P1	0.01	0.04	0.00	0.00	0.00	23	18	17	34
2527761	CD99P1	0.01	0.00	0.00	0.00	0.00	33	29	30	42
2527762	CD99P1	0.00	0.04	0.00	0.00	0.00	23	18	17	34
2527770	CD99P1	0.01	0.00	0.03	0.00	0.00	33	29	29	42
2527771	CD99P1	0.02	0.09	0.00	0.00	0.03	23	18	17	34
2527819	CD99P1	0.01	0.00	0.00	0.00	0.00	33	29	30	42
2527820	CD99P1	0.01	0.09	0.00	0.00	0.03	23	18	18	34
2527822	CD99P1	0.01	0.03	0.00	0.00	0.00	33	29	30	42
2527823	CD99P1	0.00	0.04	0.00	0.00	0.00	23	18	18	34
2527842	CD99P1	0.00	0.04	0.00	0.00	0.03	23	18	18	34
2609371	CD99	0.05	0.00	0.05	0.05	0.00	11	22	22	35
2609377	CD99	0.00	0.00	0.00	0.00	0.00	11	20	22	34
2609385	CD99	0.01	0.00	0.00	0.00	0.03	11	20	22	34
2609387	CD99	0.02	0.00	0.00	0.00	0.03	11	20	22	34
2609396	CD99	0.01	0.00	0.00	0.00	0.00	11	20	22	34
2609408	CD99	0.01	0.00	0.00	0.00	0.00	11	20	22	34
2609410	CD99	0.00	0.00	0.00	0.00	0.00	11	20	22	34
2609418	CD99	0.00	0.00	0.00	0.05	0.00	11	20	22	34
2609424	CD99	0.01	0.00	0.00	0.00	0.00	11	20	22	34
2609437	CD99	0.00	0.00	0.00	0.00	0.00	11	20	22	34
2609458	CD99	0.00	0.00	0.00	0.00	0.00	11	20	22	34
2609461	CD99	0.02	0.00	0.00	0.00	0.00	11	20	22	34
2609941	CD99	0.04	0.08	0.02	0.00	0.06	49	57	28	97
2610019	CD99	0.00	0.00	0.00	0.00	0.00	51	57	28	97
2610026	CD99	0.04	0.01	0.02	0.00	0.03	81	95	44	119
2610038	CD99	0.02	0.00	0.00	0.00	0.00	50	61	36	66
2610040	CD99	0.00	0.00	0.00	0.00	0.00	50	61	36	66
2610049	CD99	0.01	0.00	0.00	0.00	0.03	50	61	35	66
2610061	CD99	0.01	0.04	0.00	0.00	0.00	50	61	35	66
2610066	CD99	0.01	0.04	0.00	0.00	0.00	50	61	35	66
2610078	CD99	0.00	0.00	0.00	0.00	0.00	50	61	35	66
2610104	CD99	0.00	0.00	0.00	0.00	0.00	50	61	35	66
2610107	CD99	0.00	0.00	0.00	0.00	0.00	50	61	35	66
2610119	CD99	0.01	0.00	0.00	0.00	0.00	50	61	35	66

2610156	CD99	0.00	0.00	0.00	0.00	0.00	50	61	33	66
2610170	CD99	0.00	0.00	0.00	0.00	0.00	50	61	33	66
2610181	CD99	0.06	0.00	0.05	0.00	0.01	74	65	20	68

A yellow background shows CpGs in duplicated regions. A pale blue background shows CpGs in deleted regions. When the methylation ratio is higher than 0.25, the methylation ratio is shown in red letters in the patients.
